# Enhancing missense variant classification in predicted intrinsically disordered regions

**DOI:** 10.1371/journal.pone.0354365

**Published:** 2026-07-27

**Authors:** Rohan D. Gnanaolivu, Steven N. Hart

**Affiliations:** 1 Department of Quantitative Health Sciences, Mayo Clinic, Rochester, Minnesota, United States of America; 2 Department of Laboratory Medicine and Pathology, Mayo Clinic, Rochester, Minnesota, United States of America; Tokai University School of Medicine, JAPAN

## Abstract

Classifying disease-causing missense variants in intrinsically disordered regions (IDRs) remains a significant challenge, with over 25% of known deleterious variants occurring in these regions. Existing *in silico* missense variant predictors that predict variant classification generally perform better in ordered regions of the protein, limiting their effectiveness. To address this, we developed a machine learning methodology that integrates global IDR conformation (gIDRc) features from ALBATROSS, phase separation (PS) features from BioPython, and 1024-dimensional protein embeddings from ProtTransBertBFD generated for both wild-type (WT) and mutant IDR sequences. IDR boundaries were defined using the AlphaFold-RSA predictions, which identifies disordered regions based on AlphaFold2 pLDDT scores and relative solvent accessibility. Using ClinVar variant classifications as ground truth, AlphaMissense, EVE, and ESM1b were the highest scoring unsupervised *in silico* missense predictors for IDR variants. Our baseline model, using only IDR-specific features achieved competitive performance on the hold-out test set with a PR-AUC of 0.817. Critically, when these IDR features were combined with these methods we saw significant overall improvement. The AlphaMissense-Enhanced model increased its PR-AUC from 0.807 to 0.919. Similarly, ESM1b-Enhanced improved PR-AUC from 0.679 to 0.845 and EVE increased from 0.591 to 0.910. These results demonstrate the effectiveness of our enhancements for classifying missense variants in IDRs and highlight its ability to complement existing *in silico* missense predictors.

## Introduction

Missense variants are single nucleotide polymorphisms (SNPs) that result in the substitution of a single amino acid in the protein sequence. These changes can have a wide range of effects on protein function, from benign alterations to severe pathogenic consequences. For example, the Glu6Val substitution causes sickle cell disease through polymerization of deoxygenated hemoglobin, while the Glu6Lys substitution results in hemoglobin C disease, a clinically milder condition characterized by hemolytic anemia without the vaso-occlusive complications characteristic of sickle cell disease [1]. Understanding the functional impact or pathogenic potential of missense variants is essential for accurate disease diagnosis, prognosis, and therapeutic decision-making. Databases such as ClinVar [2] and Human Gene mutation database (HGMD) [3] contain curated missense variants that are classified for their role in causing disease. However, a large proportion of missense variants remain classified as variants of uncertain significance (VUS), in part because many occur at low population frequencies that, while consistent with pathogenicity under purifying selection, are insufficient alone to establish a causal role in disease, and because functional characterization of individual variants at scale remains impractical [4].

Many computational *in silico* missense predictors have been developed to aid in classifying missense variants [5–10]. These models employ machine and deep learning techniques, including supervised and unsupervised approaches, and rely on feature sets based on sequence conservation, protein secondary structure, protein stability, and physicochemical properties to assess variant pathogenicity. Many of these models are trained on human variant datasets, including population databases and clinical repositories such as ClinVar and gnomAD. A subset leverage evolutionary signals derived from cross-species sequence alignments, while PrimateAI specifically uses common variants observed in non-human primates as a proxy for benign variation, under the assumption that variants tolerated across primate species are unlikely to be pathogenic in humans [11–13]. Some *in silico* missense predictors are referred to as meta predictors, as they learn from the combined predictions of individual *in silico* tools [14–16]. The American College of Medical Genetics and Genomics (ACMG) guidelines also recognize the importance of computational missense predictors, categorizing them as supportive evidence for pathogenic (PP3) and benign (BP4) classifications [17], under the assumption that a prediction of altered function is equivalent to pathogenicity [18]. Recent predictors such as AlphaMissense [19], ESM1B [20], and EVE [7] has demonstrated strong performance from missense variants in structured regions. However, the performance in intrinsic disordered regions (IDRs) are suboptimal compared to their performance in ordered regions [19,21,22]. For example, AlphaMissense reports an average area under the curve (ROC-AUC) of 0.94 for missense variants in ordered regions but only about 0.85 for missense variants in disordered regions.

IDRs are highly abundant in the eukaryotic proteome, accounting for nearly 30% of all proteins, and play critical roles in processes such as transcriptional regulation, DNA replication, and signal transduction [23]. IDRs are protein segments that lack a stable secondary or tertiary structure and exist as dynamic ensembles. Their structural flexibility makes them challenging to study using X-ray crystallography or cryo-electron microscopy [24]. Instead, IDRs are typically identified based on their amino acid composition, and various disorder prediction algorithms have been developed to assess their propensity for disorder [25]. AlphaFold2 demonstrated a strong correlation between low-confidence predictions and intrinsic disorder. A study found that a combination of the confidence score Predicted Local Distance Difference Test (pLDDT) from AlphaFold2 and Relative Solvent Accessibility (AlphaFold-RSA) provides a robust approach for predicting disordered regions within proteins [26,27].

IDRs do not adopt a single stable structure; instead, they exist in multiple conformations, influencing their flexibility and interaction potential. Recent advances in the prediction of global protein conformation (gIDRc) can predict biophysical properties of IDRs [23], providing insight into their structural adaptability altered behavior. Beyond structural flexibility, IDRs often drive biomolecular phase separation (PS), forming dynamic membrane-less organelles that regulate cellular organization. Several methodologies exist that uses IDR-relevant biophysical and compositional features to predict PS [28,29]. Variants within IDRs can disrupt PS, leading to loss or gain of function and contributing to diseases such as neurodegeneration and cancer [22]. In addition to biophysical modeling, protein language models (pLMs) offer a powerful approach to understanding missense variant effects in IDRs. Recent studies have demonstrated that pLMs can effectively capture sequence-based features relevant to protein function, making them valuable tools for missense variant prediction [30].

To improve missense variant interpretation in IDRs, we developed a methodology specifically tailored for IDRs, integrating features predictive of gIDRc, PS, and embeddings from pLMs to enhance existing *in silico* missense predictors. Unlike existing tools, which struggle with the unique properties of IDRs, our approach leverages disorder-specific features to improve classification accuracy. By combining IDR-specific biophysical properties with unsupervised models (AlphaMissense, ESM1b, and EVE) and comparing them to models trained on ClinVar, our method demonstrates improved predictive performance. This framework enables a more accurate distinction between pathogenic and benign missense variants in disordered regions, addressing a critical gap in current computational approaches.

## Materials and methods

### Model generation

**Fig 1:**
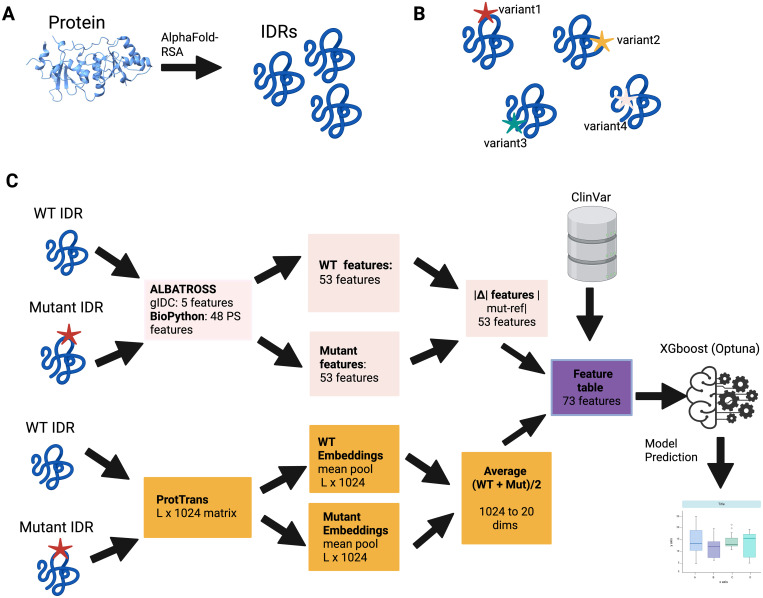
Illustration figure of the computational framework for predicting the impact of missense variants in Intrinsically Disordered Regions (IDRs). (A) Predictions from AlphaFold-RSA were used to predict the IDRs from AlphaFold structures retrived from MobiDB “prediction-disorder-alphafold” annotation, which defines disordered residues based on pLDDT scores below 70% and relative solvent accessibility above 0.581 computed over a 25-residue sliding window. (B) Missense variants were introduced into the FASTA sequence of the predicted IDR sequence after mapping the protein coordinates with the genomic coordinates listed in the ClinVar database. Different variants (variant1, variant2, variant3, variant4) represent variants occurring at various positions of the IDRs. (C) Features were extracted from both wild-type (WT) and mutant IDR sequences using two parallel pipelines. ALBATROSS (5 gIDRc features) and BioPython (48 PS features) were applied to both sequences, producing 53 scalar features per sequence. Absolute delta values (|mutant − WT|) were computed for each feature, yielding 53 variant-level perturbation features. In parallel, ProtTransBertBFD generated per-residue embeddings of dimension L × 1024 for each sequence, which were mean-pooled across residue positions to produce a single 1024-dimensional vector per sequence. The WT and mutant vectors were combined using element-wise averaging, and F-statistic feature selection retained the top 20 most discriminative embedding dimensions. All features were concatenated into a final feature table and used as input to an XGBoost classifier optimized using the Optuna framework. ClinVar pathogenicity classifications served as ground truth labels. Model performance was evaluated using ROC-AUC and PR-AUC.

To analyze the impact of missense variants in IDRs, we utilized protein coordinate predictions of disorder from AlphaFold-RSA, downloaded from MobiDB [31]. using the “prediction-disorder-alphafold” annotation category. Within MobiDB, this annotation is computed from AlphaFold2 structure predictions using two complementary disorder definitions, residues with a pLDDT confidence score below 70% are classified as disordered, and residues with a per-residue relative solvent accessibility (RSA) above 0.581, computed by DSSP and averaged over a sliding window of 25 residues, are independently classified as disordered. RSA is a normalized measure of how exposed a residue is to solvent relative to its maximum possible exposure in a fully extended peptide; high RSA values are characteristic of flexible, unstructured regions. The RSA threshold of 0.581 was selected by maximizing F1-score performance on the CAID DisProt benchmark dataset [27]. Contiguous segments of residues meeting either disorder criterion define the IDR coordinate intervals used in this study. The dataset was filtered for human proteins (NCBI Taxon ID 9606), and reference protein FASTA sequences were downloaded from UniProt using the corresponding UniProt IDs (Fig 1A). We then identified all missense variants from the ClinVar database whose protein position fell within a predicted IDR interval. For each variant, a mutant FASTA sequence was generated by introducing the amino acid substitution at the corresponding position within the predicted IDR segment of the reference sequence (Fig 1B). This approach ensured that only variants occurring within IDRs were considered for further analysis. The reference and mutant FASTA sequences were then used as inputs for feature and embedding extraction using tools ALBATROSS [23] and ProtTransBertBFD [30]. ALBATROSS is used to predict biophysical properties that can be used to infer global protein conformation from an IDR. IDR-relevant biophysical and compositional features is used to predict PS from an IDR protein sequence. ProtTransBertBFD, similar to ESM1b, employs transformer architectures pre-trained on large protein sequence databases to generate contextualized amino acid embeddings. These models learn evolutionary and biochemical patterns through self-supervised masked language modeling, enabling representation of sequence context without explicit structural or alignment information. For ALBATROSS and features that represent PS, the absolute delta change between the reference and mutant sequences was computed to quantify the structural and PS alterations caused by the variant. With ProtTransBertBFD, protein embeddings were initially generated for each amino acid across the entire IDR protein sequence input, subsequently, the mean of the embeddings across all amino acids in the sequence was calculated to create a single scalar representation for the protein sequence. This process was performed separately for the reference and mutant sequences. To capture mutation induced shifts in feature representation, the average of the reference and mutant embeddings was computed. This aggregated representation effectively summarizes the contextual changes introduced by the mutation and was used as the input feature set for downstream analysis. The extracted features from ALBATROSS, IDR-relevant compositional features and ProtTransBertBFD were then concatenated. A gradient boosting classifier (XGBoost) was trained, optimized using hyperparameter tuning with Optuna [32] and validated on a hold-out test set to assess its performance in distinguishing the impact of missense variants in IDRs (Fig 1C). The code for this study is available at https://github.com/rohandavidg/IFP-MIDR

### Data selection

The variants used in this study were downloaded from the ClinVar 2024-09-17 release, with variants located in Pfam domains filtered out as per the coordinates downloaded from University of California Santa Cruz (UCSC) resources [33]. The ClinVar VCF file was annotated with CAVA v2 [34] to determine the protein substitution from genomic variants, leveraging transcripts from NCBI and EMBL-EBI (MANE) transcripts that corresponds to UniProt protein FASTA reference. IDRs predicted by AlphaFold2-RSA were observed in 834 genes that mapped to human NCBI Taxon ID 9606 as per the annotations found in the MobiDB. The threshold used to determine if an amino acid is within a disordered region is 0.581. To align these predictions with ClinVar transcript annotations, Ensembl transcripts associated with UniProt IDs were mapped to NCBI transcripts. This process ensured that protein coordinates were consistently aligned with genomic coordinates based on transcript information from the annotated ClinVar VCF, resulting in a total of 15,999 variants located in predicted IDR regions. Based on the clinical classification in ClinVar, 85.7% of the variants in IDRs are classified as Variant of Uncertain Significance (VUS) and we retained only the variants were there pathogenic/likely pathogenic (deleterious) and benign/likely benign (neutral) variants (S1 Text).

The ClinVar classification of missense variants was grouped into three categories, which were Deleterious, Neutral and VUS. Variants labeled as ‘Likely_pathogenic’, ‘Pathogenic/Likely_pathogenic’, ‘Pathogenic|drug_response’, ‘Pathogenic’, ‘Pathogenic|other’, ‘Pathogenic/Likely_pathogenic|other’, ‘Likely_pathogenic|other’, and ‘Likely_pathogenic/Likely_risk_allele’ were classified as Deleterious, while those categorized as ‘Likely_benign’, ‘Benign’, and ‘Benign/Likely_benign’ were classified as Neutral, and the remaining was categorized as VUS. Further refinement to include only genes with Ensembl transcripts that could be mapped to an orthogonal RefSeq transcript reduced the dataset to a total of 2,203 variants (S1 Text).

To evaluate the performance of existing predictors for variants found in predicted IDRs, we utilized data from dbNSFP v4.8 [35], which includes a comprehensive set of *in silico* missense prediction scores from 56 predictors, including un-supervised models AlphaMissense, ESM1b, and EVE. Intersection of the filtered ClinVar IDR dataset with dbNSFP v4.8 was performed to retrieve available *in silico* prediction rankscores for each variant. Variants absent from dbNSFP, predominantly due to isoform or transcript mismatches between the ClinVar MANE transcript annotations and the transcripts referenced in dbNSFP, were excluded. This filtering reduced the dataset from 2,203–2,104 missense variants with known pathogenicity classifications spanning 290 genes (S1 Table). In total, the dataset comprised of 316 variants classified as deleterious and 1788 variants classified as neutral. Missense variants classified as VUS were not used for model development or evaluation. The predictive performance of each *in silico* missense predictor was assessed using its normalized rank scores available in the dbNSFP database. Performance metrics for all 23 dbNSFP predictors were computed on the full labeled dataset of 2,104 variants, as these models were not trained by the authors and are therefore not subject to overfitting concerns with respect to our data split.

### Feature generation

Features such as radius of gyration, end-to-end distance, asphericity, and prefactor predicting gIDRc were generated using ALBATROSS within Sparrow v0.2.3 for both mutant and WT using the input FASTA sequences representing IDRs. Features predicting PS were evaluated using IDR-relevant compositional features. These features include local hydrophobicity patterns that are relevant to IDR compaction and binding, charge distribution that is important for electrostatic interactions in IDRs, low complexity regions which is known to be associated with both phase separation and IDR functions and aromatic and aliphatic content, which is relevant to π-π and hydrophobic interactions. In total, there were 15 features for wild-type (WT) and mutant sequences. To incorporate sequence-level representations, we used the pLM ProtTransBertBFD to generate per-residue embeddings for both WT and mutant IDR sequences. For a sequence of length L, ProtTransBertBFD produces an L × 1024 embedding matrix, where each row corresponds to the 1024-dimensional representation of one amino acid position. Mean pooling was then applied across all L residue positions to yield a single 1024-dimensional sequence-level vector per sequence, a standard approach for obtaining fixed-length representations from transformer-based protein language models. This process was performed independently for the WT and mutant sequences, producing one 1024-dimensional vector for each. These combined features of gIDRc, IDR-relevant compositional features, and deep learning-based embeddings provided a comprehensive representation of the potential deleterious impact of missense variants in IDRs.

### Feature preprocessing

The features generated by ALBATROSS (gIDRC) and BioPython (PS) for both mutant and WT IDR sequences were used to compute the delta change, capturing the difference between the two sequence states. The absolute value of these deltas were then calculated to ignore the direction of change and quantify the magnitude of change independent of direction. To evaluate different approaches for combining WT and mutant embeddings into a single variant-level representation, the 1024-dimensional sequence-level embeddings generated by ProtTransBertBFD for the mutant and WT sequences were calculated using four different combination strategies, which were L1 (absolute difference), L2 (Euclidean distance), average (mean of embeddings), and Hadamard product (element-wise dot product). The four embedding combination strategies (L1, L2, average, Hadamard) were applied exclusively to the ProtTransBertBFD embeddings and not to the gIDRc or PS features.

Let E_m_ and E_w_ represent the embeddings from the mutant and WT, respectively. We define Hadamard, Average, L1 and L2 as follows:


$$\begin{array}{c} Hadamard\;\left(E_{m},\;E_{w}\right)=f\left(E_{m}\right).\;f\left(E_{w}\right) \end{array}$$

$$\begin{array}{c} Average\;\left(E_{m},\;E_{w}\right)=\;\frac{f\left(E_{m}\right)+f(E_{w})}{\text{2}} \end{array}$$

$$\begin{array}{c} L\text{1}\;\left(E_{m},\;E_{w}\right)=|f\left(E_{m}\right)-f\left(E_{w}\right)| \end{array}$$

$$\begin{array}{c} L\text{2}\left(E_{m},\;E_{w}\right)={\left|f\left(E_{m}\right)-f\left(E_{w}\right)\right|}^{\text{2}} \end{array}$$

The gIDRc and PS absolute delta features are low-dimensional scalar quantities (5 and 48 features respectively) that directly encode the biophysical perturbation caused by the amino acid substitution and require no dimensionality reduction. Each embedding combination strategy produces a 1024-dimensional vector, necessitating feature selection prior to model training. F-statistic feature selection (ANOVA F-test, SelectKBest, scikit-learn) was applied to retain the top 20 embedding dimensions most associated with variant pathogenicity. In the embedding comparison experiment (Fig 4), the selector was fitted within each cross-validation (CV) fold on training fold rows only, and the selected indices were applied to the held-out validation fold without refitting. In the hyperparameter optimization pipeline, the selector was fitted once on the full 80% training partition after the 80/20 split, and the same selected indices were applied to transform the held-out test set. In both cases the test data was never accessed during feature selection. All features were z-score standardized using a StandardScaler fitted on training data only and applied to the test data without refitting.

### Model creation

The features derived from ALBATROSS, PS, and the ProtTransBertBFD embeddings were generated for a total of 2,104 variant, comprising 316 deleterious and 1788 neutral variants. These features were concatenated to form a comprehensive dataset for model evaluation. To assess predictive performance, we compared different model predictors, which were, Random Forest, Multi-Layer Perceptron (MLP), Naïve Bayes, and XGBoost classifiers using scikit-learn v0.24.2. We employed 10-fold CV to evaluate model performance, computing ROC-AUC and PR-AUC as primary metrics. A random stratified CV was employed, which maintains class proportions within each fold and reflects performance on variants from genes present in the training set. To ensure there was no data leakage, we computed pairwise sequence identity between sequences in the training and test sets and found that 0% of sequences in the test set had 100% identity with those in the training set.

### Hyperparameter optimization

We employed the Optuna framework to optimize the hyperparameters of the XGBoost model. The dataset was split into an 80:20 ratio using random stratified splitting, where 80% of the data (1,683 variants) was used in 10-fold stratified CV to determine the optimal hyperparameters, with the stratification ensuring that there is even distribution of classes within each fold. Prior to hyperparameter optimization (HPO), F-statistic feature selection was applied on the training partition only to reduce the embedding dimensionality from 1,024–20, as described in the Feature Preprocessing section. A total of 150 optimization trials were conducted, with the objective of maximizing the mean PR-AUC across the 10-fold stratified CV (StratifiedKFold). Once the optimal hyperparameters were identified, the final tuned model was applied to the hold-out test set comprising the remaining 20% of the data (421 variants) to evaluate its performance on unseen data.

### Combination with AlphaMissense, EVE and ESM1B

To evaluate the complementary value of existing *in silico* predictors, the XGBoost model was retrained, and HPO was performed with AlphaMissense, EVE and ESM1b added separately as additional features to assess their impact on predictive performance. Additionally, two combined models were evaluated, a standalone combined model incorporating only the three predictor rankscores without any IDR specific features (AlphaMissense + ESM1b + EVE standalone), and a full combined model incorporating all three predictor rankscores together with the complete IDR specific feature set (AlphaMissense + ESM1b + EVE + IDR specific features). For all six models, the dataset was split into an 80:20 ratio, where 80% of the data was used for 10-fold stratified CV to determine the optimal hyperparameters and the remaining 20% was used as the hold-out test set. F-statistic feature selection was applied on the training partition only prior to optimization, as described in the Feature Preprocessing section. A total of 150 Optuna optimization trials were conducted per model, with the objective of maximizing mean PR-AUC across 10-fold stratified cross-validation within the training partition. Once optimal hyperparameters were identified for each model, the final model was trained on the full training partition and evaluated on the held-out test set.

### ClinVar review status classification

The hold-out test set was stratified based on ClinVar’s review status, using its star rating system. Variants labeled as “no assertion criteria provided” or “criteria provided, single submitter” were grouped under the single-star category. Variants with “criteria provided, multiple submitters, no conflicts” were categorized as two stars, while those “reviewed by expert panel” were assigned to the three-star category. This stratification resulted in 292 variants in the one-star category, 100 in the two-star category, and 29 in the three-star category. Within each star category the ROC-AUC and the PR-AUC were calculated, along with its confidence intervals (CI).

### dbNSFP *in silico* model comparison

To evaluate whether the proposed methodology provides complementary predictive value in regions where existing missense predictors fail in classifying IDR variants, we performed a variant-level misclassification analysis across all 56 *in silico* predictors available in dbNSFP v4.8. For each variant in the hold-out test set, the misclassification rate was computed as the proportion of dbNSFP predictors that assigned an incorrect classification relative to the ClinVar ground truth label. Variants were then ranked by this misclassification rate to identify those most consistently challenging for existing tools. We subsequently assessed the performance of the enhanced model on this difficult subset, allowing us to determine whether IDR-specific biophysical features provide additional discriminative power precisely where sequence-based predictors struggle. Critically, variant selection for this analysis was based exclusively on the collective error rate of existing dbNSFP predictors.

### Validation with GnomAD Allele Frequency

To evaluate the results from the test data, we evaluated the relationship between enhanced model scores and population allele frequencies. Predictions from AlphaMissense Enhanced, ESM1b Enhanced, and EVE Enhanced models were compared against GnomAD v2.1.1 exome allele frequencies for 421 missense variants with available population frequency data. Under the assumption that truly deleterious variants are subject to purifying selection and thus occur at lower population frequencies, we expected pathogenic predictions to correlate with lower GnomAD allele frequencies.

### Statistical analysis

The association between each gIDRc feature (asphericity, radius of gyration, end-to-end distance, scaling exponent, and prefactor) and variant pathogenicity was assessed using the Mann-Whitney U test, as it is a non-parametric test suitable for comparing two groups. Individual predictive performance of each gIDRc feature was quantified using ROC-AUC and PR-AUC. The performance of all 56 dbNSFP *in silico* predictors on IDR variants was evaluated using ROC-AUC and PR-AUC as primary metrics, with 95% confidence intervals generated by bootstrap resampling over 1,000 iterations.

Feature selection was performed using the ANOVA F-statistic (SelectKBest, scikit-learn) to identify the 20 most discriminative dimensions from the 1,024-dimensional ProtTransBertBFD embedding vector. The F-statistic tests whether the mean embedding value for a given dimension differs significantly between deleterious and neutral variants, ranking dimensions by their univariate association with the binary pathogenicity label. To prevent data leakage, the F-statistic selector was fitted exclusively on training data in all experiments, within each CV fold during the embedding comparison experiment

Comparison of embedding combination strategies (L1, L2, Average, Hadamard) and classifiers (XGBoost, Random Forest, MLP, Naïve Bayes) was performed using the Kruskal-Wallis test to assess overall group differences, followed by Dunn's post hoc test with Bonferroni correction for pairwise comparisons. Both ROC-AUC and PR-AUC were used as performance metrics throughout.

Pairwise comparisons between standalone *in silico* predictors (AlphaMissense, ESM1b and EVE) and their corresponding enhanced models were performed using the Mann-Whitney U test (two-sided) applied to the 10-fold CV fold scores and its statistical significance was reported.

## Results

### Evaluation of dbNSFP predictors with functions

**Fig 2:**
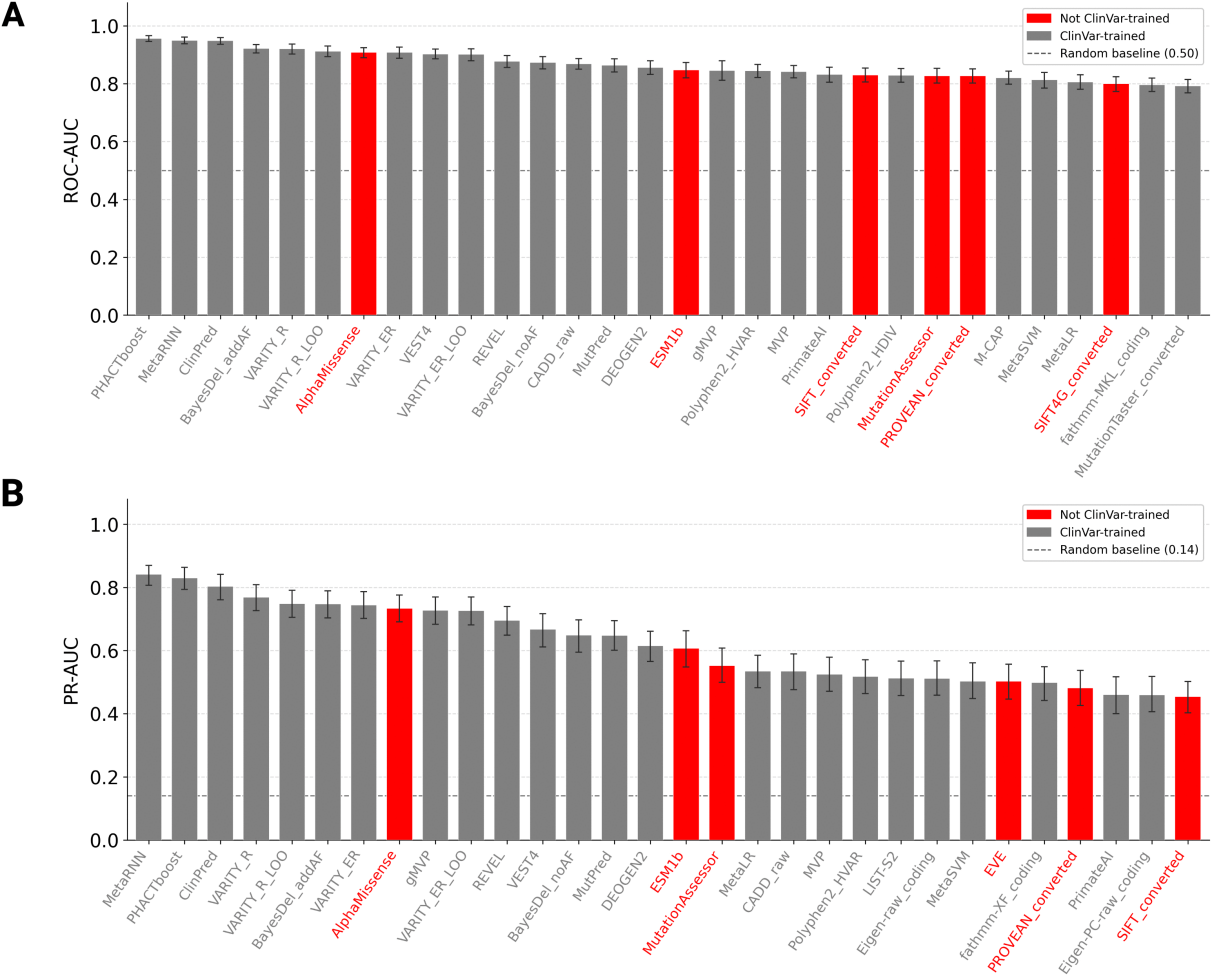
Performance metrics of in silico missense predictors on variants found in IDR regions as per AlphaFold-RSA predictions (A) The ROC-AUC performance of the top 30 in silico missense predictors listed in the dbNSFP v4.8 databases, ranked by performance. Error bars represent 95% confidence intervals from bootstrap resampling over 1,000 iterations. The random baseline (ROC-AUC = 0.50) is shown as a dashed line. Predictors shown in red were not trained on ClinVar variant classifications. Predictors in grey incorporate ClinVar-derived labels in their training. (B) PR-AUC performance of the top 30 in silico missense predictors listed in the dbNSFP databases, ranked by performance. The random baseline PR-AUC of 0.14 reflects the positive class prevalence in the dataset. Predictors shown in red were not trained on ClinVar variant classifications. Predictors in grey incorporate ClinVar-derived labels in their training.

A significant number of *in silico* missense predictors are trained on ClinVar and HGMD datasets, which exhibit substantial overlap in their training data and the data used in this study (Fig 2). However, 23 models in the dbNSFP database are not trained on ClinVar and HGMD. Among these, when focusing on variants located in predicted IDR regions, AlphaMissense, an un-supervised model, demonstrated the highest predictive performance with an ROC-AUC of 0.907 (95% CI:0.905–0.909) (Fig 2A) and PR-AUC of 0.733 (95% CI:0.690–775) (Fig 2B), followed by ESM1b with an ROC-AUC of 0.847 (95% CI: 0.819–0.872) and PR-AUC of 0.606 (95% CI:0.548–0.662). Additionally, EVE, the other unsupervised autoencoder model using sequence conservation, achieved an ROC-AUC of 0.715 (95% CI: 0.679–0.751) and PR-AUC of 0.50 (95% CI:0.446–0.556), highlighting its moderate performance for missense classification in predicted IDRs. These results, derived from ClinVar-labeled variants located in AlphaFold-RSA predicted disordered regions, suggest that AlphaMissense currently represents the state-of-the-art benchmark for predicting deleterious missense variants in IDRs (S1 Text).

### Association of global IDR conformation with protein function

**Fig 3:**
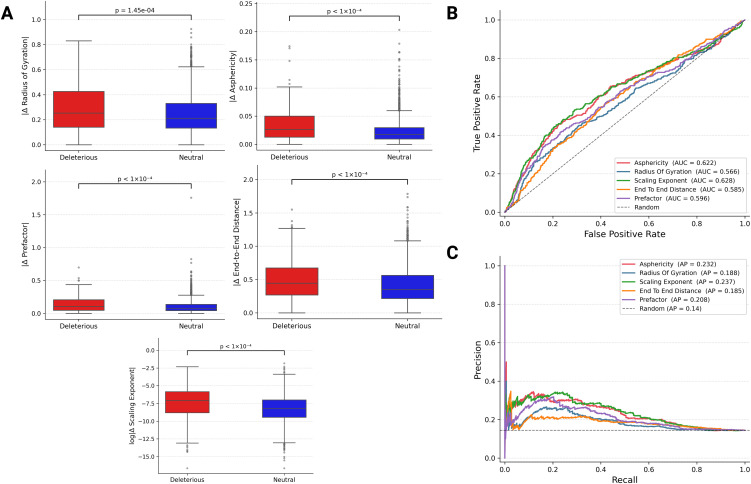
Individual predictive performance of gIDRc features for IDR missense variant classification. (A) Association of the absolute change in Radius of Gyration, Asphericity, Scaling Exponent, Prefactor, End to End distance between WT and mutant IDR sequences, stratified by ClinVar pathogenicity classification (deleterious in red, neutral in blue). Square root transformation was applied to radius of gyration, asphericity, prefactor, and end-to-end distance for visualization. Log transformation was applied to scaling exponent. All statistical comparisons were performed on untransformed values using the Mann-Whitney U test. (B) ROC-AUC curves for each individual gIDRc feature. (C) Precision-recall curves for each individual gIDRc feature. The random baseline PR-AUC of 0.14 reflects the positive class prevalence in the dataset.

Using a Mann-Whitney U test, we evaluated the association between protein function and the absolute change between WT and mutant sequences for all five gIDRc features, which are, radius of gyration, end-to-end distance, asphericity, and prefactor. All five features demonstrated a statistically significant association with pathogenicity (p < 0.05), indicating that deleterious variants induce greater perturbation to global IDR conformation than neutral variants. For visualization, square root transformation was applied to asphericity, radius of gyration, end-to-end distance, and prefactor, while a log transformation was applied to scaling exponent due to its more heavily right-skewed distribution. All statistical tests were performed on the original untransformed absolute delta values (Fig 3A). Further evaluation of their predictive performance based on the ROC-AUC metric, revealed that the absolute change in scaling exponent had the highest ROC-AUC of 0.628, followed by the remaining four features, all of which exceeded the random baseline of 0.50 (Fig 3B), highlighting their potential relevance in impact assessment. PR-AUC was additionally computed for each feature to account for class imbalance. All five features exceeded the random baseline PR-AUC of 0.14, with scaling exponent achieving the PR-AUC of 0.237, followed by asphericity with a PR-AUC of 0.232 (Fig 3C). The consistent ranking of scaling exponent as the most discriminative gIDRc feature across both ROC-AUC and PR-AUC suggests that mutation-induced changes to the scaling behavior of the IDR polymer chain are particularly informative for distinguishing pathogenic from neutral variants

### Evaluation of embedding combination method and model performance

**Fig 4:**
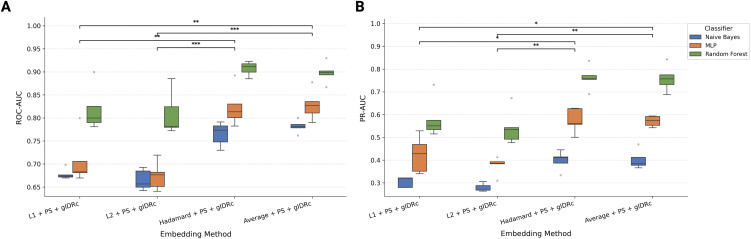
Performance comparison of multiple protein embedding combination methods using multiple machine learning models to predict protein function on classified variants found in ClinVar in predicted IDR regions. (A) ROC-AUC and (B) PR-AUC distributions across 10-fold cross-validation for four embedding combination methods (L1, L2, Hadamard, Average) combined with gIDRc and PS features, evaluated using Random Forest, MLP, and Naïve Bayes classifiers. Significance brackets indicate pairwise comparisons with Bonferroni-corrected p-values from Dunn's post hoc test following Kruskal-Wallis testing (* p < 0.05, ** p < 0.01, *** p < 0.001).

We evaluated four different embedding combination methods (L1, L2, average, and Hadamard) across four different prediction models (XGBoost, Random Forest, Naïve Bayes, and MLP) to predict protein function Using mean ROC-AUC and PR-AUC across 10-fold stratified CV. Statistical comparisons across embedding methods were performed using the Kruskal-Wallis test followed by Dunn's post hoc test with Bonferroni correction. We found both PR-AUC and ROC-AUC, XGBoost and Random Forest consistently outperformed MLP and Naïve Bayes across all four embedding methods. The Average and Hadamard combination strategies achieved the highest performance for both XGBoost and Random Forest. For ROC-AUC, Hadamard + PS + gIDRc and Average + PS + gIDRc with both XGBoost and Random Forest reached values of 0.89–0.91, which significantly outperforming L1 (p < 0.01) and L2 (p < 0.001) combinations(Fig 4A and S1 Table). Similarly, for PR-AUC, the same pattern held, with Hadamard and Average combinations significantly outperforming L1 (p < 0.05) and L2 (p < 0.01) (Fig 4B). The L2 combination consistently showed the weakest performance across both metrics and all classifiers, while L1 showed intermediate performance. Naïve Bayes and MLP performed substantially below XGBoost and Random Forest across all embedding strategies. Kruskal-Wallis testing confirmed a significant effect of embedding method on both ROC-AUC and PR-AUC (p < 0.001). Based on these results, the Average embedding combination with XGBoost was selected for downstream hyperparameter optimization, as it demonstrated performance equivalent to Hadamard while providing a more interpretable representation of the shared IDR sequence context between WT and mutant sequences. The default hyperparameters for XGBoost included learning_rate = 0.3, n_estimators = 100, max_depth = 6, gamma = 0, and subsample = 1.0. Given its robust performance, we selected XGBoost trained on the average embedding combination, along with features from gIDRc and PS, as the final model for further optimization.

### Hyperparameter optimization (HPO)

Using the Optuna framework, we further optimized the XGBoost model through 150 trials, aiming to maximize the mean PR-AUC across 10-fold randomly stratified CV within the 80% training partition. Each trial represents a new set of hyperparameters that is tested. The first trial, representing a randomly sampled hyperparameter set, achieved a mean CV PR-AUC of 0.688. The best trial achieved a mean CV PR-AUC of 0.744, representing an absolute improvement of 0.056 over the initial random parameter set and a median trial PR-AUC of 0.735 across all 150 trials. The lower CV PR-AUC relative to the embedding comparison experiment reflects the more conservative evaluation design as HPO is performed on the 80% training partition only, with fewer deleterious variants available per fold compared to the full-dataset CV used for model selection

When applied to the hold-out test set, which comprised of 421 (63 deleterious, 358 neutral), the optimized baseline model achieved a PR-AUC of 0.807 (95% CI 0.711–0.876) and ROC-AUC of 0.932. The final set of optimal hyperparameters identified included: n_estimators = 427, max_depth = 9, learning_rate = 0.024, colsample_bytree = 0.621, subsample = 1.000, gamma = 0.175, min_child_weight = 1, reg_alpha = 1.126, and reg_lambda = 8.948. Correlation analysis between hyperparameter values and mean CV PR-AUC across all 150 trials revealed that gamma, reg_alpha, and min_child_weight showed the strongest associations with model performance. lower values of these regularization parameters were associated with higher PR-AUC, suggesting the model benefits from reduced regularization constraints and increased tree-splitting flexibility in this dataset (S1 Text).

### Feature evaluation

Utilizing SHapley Additive exPlanations (SHAP) [36] to assess feature importance in the optimized XGBoost model revealed that the embeddings combined via the average method between the WT and mutant were the most influential (S1 Text). The analysis demonstrated that PLM embeddings from ProtTransBertBFD combined via the average strategy were the most influential features overall. The highest ranked feature was embedding dimension 631 (ProtTrans_dim_631), achieving a SHAP importance value of approximately 0.089. Dimension 631 refers to the original index within the 1,024-dimensional ProtTransBertBFD embedding space that was retained after F-statistic feature selection and ranked highest by SHAP analysis, indicating that this specific region of the protein language model embedding space is the single most discriminative feature for distinguishing pathogenic from neutral IDR variants. PS features from BioPython and gIDRc features from ALBATROSS were prominently distributed throughout the importance hierarchy, with several features from each category ranking within the top 20 most influential predictors. The balanced representation of features from ProtTransBertBFD, embedding dimensions, PS features, and gIDRc features within the top-ranked predictors validates our integrative approach, demonstrating that protein language model embeddings, phase separation propensity, and global IDR conformation features each contribute distinct yet complementary information for predicting missense variant pathogenicity in intrinsically disordered regions.

### Improvement with AlphaMissense, EVE and ESM1b

As noted above, AlphaMissense, ESM1b and EVE were the highest performing unsupervised classification models. To improve these metrics, we retrained the XGBoost model and optimized its hyperparameters to create an “Enhanced” version. Enhanced model creation begins with one of AlphaMissense, ESM1b or EVE. Next, IDR-relevant compositional features, gIDRc and the average of the embeddings between the WT and mutant are added. The dataset was split into 80% training data (1,683 variants), where 10-fold random stratified CV was performed, and 20% (421 variants) was reserved as a hold-out test set.

**Fig 5:**
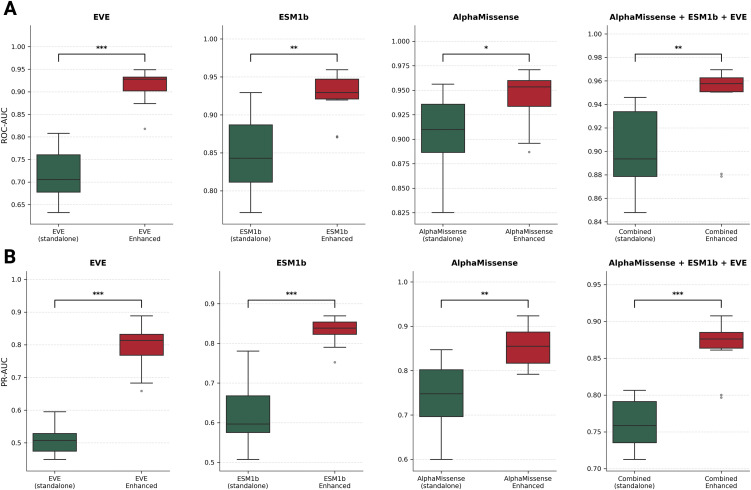
Performance of enhanced models compared to standalone in silico predictors under random stratified cross-validation (10 folds). (A) ROC-AUC and (B) PR-AUC distributions comparing standalone predictors (green) to Enhanced models incorporating the same predictor as an additional feature alongside gIDRc, PS, and ProtTransBertBFD average embeddings (red). Comparisons are shown for EVE, ESM1b, AlphaMissense, and the combined model incorporating all three predictors simultaneously (AlphaMissense + ESM1b + EVE). Statistical significance was assessed using the Mann-Whitney U test (* p < 0.05, ** p < 0.01, *** p < 0.001). No multiple testing was done.

Under random stratified evaluation, all three enhanced models significantly outperformed their respective standalone predictors. EVE incorporation as a feature also demonstrated significant performance gains. The EVE-enhanced model achieved a mean PR-AUC of 0.792 in 10-fold random stratified CV, substantially outperforming standalone EVE (PR-AUC: 0.506; 95% CI: 0.475–0.537; p < 0.001). Hold-out test validation showed EVE-enhanced achieving an PR-AUC of 0.910 and ROC-AUC of 0.966. (Fig 5A). Optimal hyperparameters were n_estimators = 466, max_depth = 10, learning_rate = 0.029, colsample_bytree = 0.863, subsample = 0.602, gamma = 0.877, min_child_weight = 1, reg_alpha = 0.38, and reg_lambda = 7.66 (S1 Table).

ESM1b integration demonstrated equally substantial performance enhancement. The ESM1b-enhanced model achieved a mean PR-AUC of 0.831 in 10-fold random stratified CV from the training set, significantly outperforming standalone ESM1b (PR-AUC: 0.622; 95% CI: 0.561–0.682; p < 0.001). The 35.19% increase in PR-AUC substantially enhances deleterious variant detection capability. Hold-out test validation confirmed superior performance with PR-AUC of 0.845 and ROC-AUC of 0.955 (Fig 5B). Optimal hyperparameters included: n_estimators = 330, max_depth = 10, learning_rate = 0.077, colsample_bytree = 0.772, subsample = 0.505, gamma = 0.624, min_child_weight = 3, reg_alpha = 1.68, and reg_lambda = 5.14.

The AlphaMissense-enhanced model achieved a mean PR-AUC of 0.855 representing a significant improvement over standalone AlphaMissense (PR-AUC: 0.740, 95% CI: 0.680–0.797; p = 0.003), and hold-out test PR-AUC of 0.919 and ROC-AUC of 0.982. The 16.78% improvement in PR-AUC is clinically significant as it directly measures enhanced detection of deleterious variants (Fig 5C). This performance rivals top *in silico* missense predictors trained on ClinVar variants, including MetaRNN (PR-AUC: 0.841, 95% CI: 0.806–0.869) and PHACTboost (PR-AUC: 0.830, 95% CI: 0.793–0.863). Optimal hyperparameters were determined through Optuna optimization: n_estimators = 390, max_depth = 3, learning_rate = 0.068, colsample_bytree = 0.83, subsample = 0.595, gamma = 1.50, min_child_weight = 1, reg_alpha = 1.12, and reg_lambda = 1.192.

The combined standalone model incorporating all three predictor rankscores without IDR specific features achieved a hold-out test PR-AUC of 0.833 and ROC-AUC of 0.939, confirming that the three existing tools together capture substantial predictive signal. However, the full combined model of AlphaMissense + ESM1b + EVE, along with IDR specific features, achieved the highest overall performance with mean CV PR-AUC of 0.864 ± 0.035 (p < 0.001 vs combined standalone) and hold-out test PR-AUC of 0.916 and ROC-AUC of 0.981, demonstrating that biophysical features contribute complementary discriminative information beyond that captured by any combination of existing *In Silico* based tools.

### Model performance on ClinVar variants based on review status

Overall, the model achieved an AUC of 0.852 and an accuracy of 0.902 on a hold-out test set comprising 421 variants. When stratified by ClinVar review categories, the model achieved an AUC of 0.81 (95% CI: 0.727–0.877) for the 292 variants in the one-star category, 0.977 (95% CI: 0.945–0.997) for the 100 variants in the two-star category, and 0.892 (95% CI: 0.75–1.00) for the 29 variants in the three-star category (S1 Text).

### *In Silico* missense predictor comparison

Our evaluation of the 20 most difficult-to-classify variants from the 421-variant random stratified hold-out test set revealed that these variants had a mean misclassification rate of 75.4% across all 56 dbNSFP predictors, representing cases where the majority of existing tools fail regardless of variant class. Despite this collective difficulty, our evaluation of the model’s performance on the test dataset demonstrated its ability to correctly classify several variants that were misclassified by the majority of dbNSFP predictors. For example, the likely pathogenic variants R573C in *FGA* and A654V in *HIF1A* were incorrectly predicted as neutral by most existing tools, including AlphaMissense, while our model accurately identified them as deleterious. Conversely, likely benign variants such as T319M and P869S in *GLI3*, and S186Y in *BRCA1*, which had the three highest misclassification rates among all 56 dbNSFP predictors (86.0%, 84.2%, and 82.5% respectively), whereas our model correctly classified them as neutral (S1 Text). We note that this analysis characterizes the performance of our method on variants that are collectively challenging for the field and includes cases where our method also fails. The selection criterion is based exclusively on the error rate of existing predictors and not on performance of the method.

### Validation with GnomAD Allele frequency

The analysis revealed clear stratification patterns consistent with purifying selection acting on deleterious variants. Pathogenic variants were predominantly found at higher prediction scores (>0.6) and lower allele frequencies (<10 ⁻ ⁴), while benign variants occupied lower prediction scores and were distributed across a broader range of population frequencies (S1 Text). Benign variants received lower prediction scores and were distributed across a broader range of gnomAD Exomes allele frequencies. The concentration of pathogenic variants at low population frequencies provides orthogonal support for model predictions independent of ClinVar training labels, as variants predicted as deleterious are appropriately depleted from the general population under negative selection. The vast majority of variants in the dataset were rare (gnomAD Exomes AF < 10 ⁻ ³), reflecting the nature of ClinVar-curated clinical variants rather than common population polymorphisms.

## Discussion

The relatively lower predictive performance of *in silico* missense classification in IDRs, compared to structured domains is well-documented [19,21]. This discrepancy may stem from the fact that most known pathogenic missense variants reside in ordered protein regions. Due to their lack of well-defined secondary structures, low evolutionary conservation, and highly dynamic nature, IDRs present significant challenges for accurate prediction of missense classification. Many computational models are overfit to structured regions, leading to biased predictions that underperform for disordered region variants. Given these challenges, we developed a methodology that utilizes biophysical properties, specific to disordered regions that enhance the classification of missense variants within IDRs.

Traditional *in silico* missense classification predictors predominantly rely on sequence conservation-based features, assessing mutation tolerance in highly conserved genomic regions and their impact on secondary structure. Even though 15–20% of IDRs are in regions with low sequence conservation, they are generally less conserved than structured domains and exhibit greater tolerance to variants [37]. Unlike ordered protein domains, IDRs lacks stable conformation, can adopt multiple structural states. This conformational heterogeneity arises from a flat energy landscape, where multiple structural conformations exist at similar energy levels rather than converging to a single low-energy folded state. This dynamic behavior makes IDRs challenging to model using conventional structure-based computational approaches that assume a single, energetically favorable conformation. Despite this, IDRs play essential roles in protein function, with approximately 25% of disease-associated variants localized within these regions [22].

The functional relevance of an IDR is closely linked to its conformational dynamics, where both long-range and short-range interactions influence its flexibility [38]. As a result, features that predict gIDRc within IDRs can be directly associated with protein function. Moreover, IDRs are integral to PS, a physicochemical process that enables the formation of biomolecular condensates. Missense variants within IDRs can disrupt key properties influencing PS, leading to neurodegenerative disorders such as Alzheimer’s and Parkinson’s disease [39]. Therefore, features predictive of PS propensity hold functional relevance in pathogenicity assessments.

PLMs like ProtTransBertBFD, combined with biophysical predictors such as Albatross and IDR-relevant compositional features, offer a powerful approach to capturing biophysical information embedded within protein sequences, providing valuable insights into predicted consequences. The optimal method for integrating embeddings from WT and mutant sequences remains system dependent. In this study, we found that the Hadamard product and average embedding combination methods outperformed L1 and L2 distance-based approaches, demonstrating their effectiveness in assessing the impact of missense variants in IDRs.

Recent studies have shown an association between changes in PS and overall protein function [22]. Our findings further highlight that alterations in gIDRc also contribute significantly to predictions, though it is not the sole predictor. Notably, we demonstrate that gIDRc, PS, and protein sequence embeddings create a robust framework for predicting the consequences of missense variants in IDRs. This approach significantly outperforms leading *in silico* missense models, including AlphaMissense, ESM1b and EVE.

Moreover, incorporating AlphaMissense, ESM1b, or EVE as additional features further enhances predictive accuracy, suggesting that gIDRc, PS, and protein embeddings provide independent and complementary information. Feature importance analysis using SHAP further supports this conclusion, revealing that gIDRc, PS, and protein embeddings rank among the top predictive features, underscoring their critical role in classification of IDR-associated missense variants. The improvement in prediction classification performance is now comparable to top *in silico* missense classification methods trained directly on ClinVar annotations.

Despite these advancements, our study has certain limitations. The selection of genes and variants was based on AlphaFold-RSA disorder predictions which, while effective in distinguishing ordered from disordered regions, are not without prediction errors. To mitigate the inclusion of variants within structured domains, all variants overlapping known Pfam domains were also excluded. However, this filtering also reduced the number of variants available for analysis and constrained our ability to validate missense variants in experimentally confirmed IDRs from DisProt within the hold-out test set. A further limitation is revealed by the performance gap between random stratified and gene-stratified evaluation, which indicates predictive performance under random splitting reflects gene-level biophysical properties encoded by gIDRc and PS features rather than purely variant-level perturbation signals. Sequence-based predictors such as AlphaMissense, ESM1b, and EVE generalize more effectively to unseen genes, as they capture evolutionary and structural context that is not dependent on gene identity. IDR specific features provide complementary discriminative signal even in the cross-gene setting, but the attenuation of performance under gene-stratified evaluation underscores the importance of this evaluation strategy as a more stringent and biologically appropriate benchmark, and highlights the need for larger, more genetically diverse IDR variant datasets to improve cross-gene generalization.

More broadly, the sparsity of high-confidence, experimentally annotated IDR variants presented a challenge for robust model training and generalizability, as well as the ability to capture the mechanistic diversity of IDR-associated variants. To address this, future work should incorporate semi-supervised learning frameworks that leverage both labeled and unlabeled IDR variant data to enhance representation learning, as well as generative models to simulate biologically plausible variants for training augmentation. Furthermore, there remains limited understanding of how IDR variants contribute to post-translational modifications, cellular signaling, and structural changes that drive oligomerization or self-assembly. Further investigations are necessary to fully elucidate the consequences of IDR associated missense variants.

While computational predictors have significantly improved missense variant classification, major limitations persist in their ability to assess IDRs. Traditional models overemphasize evolutionary conservation and structural stability, resulting in biased predictions that fail to capture the complexity of IDRs. Our model introduces an IDR-specific framework that integrates predictions of global conformation changes, phase separation dynamics, and deep learning-based embeddings to refine missense variant classification in IDRs. By addressing the shortcomings of existing approaches, this study advances the accurate classification of IDR variants and enhances our understanding of their role in disease. The code for this study is available at https://github.com/rohandavidg/IFP-MIDR

## Supporting information

S1 Text Supplementary methods, results, and figures. This file contains expanded methodological details, additional performance results, and supplementary figures S1 through S6.
(PDF)

S1 Table Supplementary tables S1–S6. A single, multi-sheet workbook containing all supplementary tables for model feature validation and performance metrics.
(XLSX)
