# Supplementary material for: Enhancing missense variant classification in predicted intrinsically disordered regions
Source: PLoS One. 2026 Jul 27;21(7):e0354365. doi: 10.1371/journal.pone.0354365 (PMC13405113; doi:10.1371/journal.pone.0354365)
Supplement: S1 Text — This file contains expanded methodological details, additional performance results, and supplementary figures S1 through S6. (PDF) [file pone.0354365.s001.pdf]

Supplementary Methods

Gene-split training and testing

To evaluate the cross-gene generalization and evaluate whether the enhanced predictive performance reflects variant-level biophysical signal rather than gene-level patterns learned during training, a gene-stratified evaluation was performed alongside the primary random stratified analysis. The dataset was partitioned using GroupShuffleSplit, ensuring all variants from a given gene were assigned exclusively to either the training or held-out test set, preventing the model from learning gene-specific sequence or biophysical properties that could artificially inflate performance on test variants from the same genes. This partitioning yielded a training set of 1,852 variants (1,612 neutral, 240 deleterious) from 232 genes and a held-out test set of 381 variants (349 neutral, 32 deleterious) from 58 genes not represented in training. We retrained the XGBoost model by incorporating AlphaMissense, ESM1b, and EVE predictions as additional features in separate experiments. Each external predictor was integrated individually with our baseline feature set to assess its specific contribution to variant classification accuracy. To prevent data leakage from variants within the same gene, the dataset was partitioned using a gene-based splitting strategy implemented through the Optuna optimization framework, ensuring that all variants from a given gene were assigned exclusively to either the training or test set, but never both. This approach prevents the model from learning gene-specific patterns during training that could artificially inflate performance on test variants from the same genes. The gene-based partitioning resulted in 1,852 variants for training and 252 variants for testing. HPO employed the Optuna framework across 150 optimization trials, with 10-fold GroupKFold cross-validation performed exclusively on the training set while maintaining gene-based separation throughout the cross-validation process to prevent information leakage during model selection. The objective function

maximized PR-AUC during cross-validation. Following optimization, final models were evaluated on the held-out test set, with performance confidence intervals calculated through bootstrap resampling over 1,000 iterations to ensure robust statistical assessment.

Supplementary Results

Gene-split training and testing evaluation

The baseline model achieved a gene-stratified test PR-AUC of 0.181 and ROC-AUC of 0.756, compared to PR-AUC 0.817 and ROC-AUC 0.932 under random stratified evaluation. The gene-stratified test set contained a lower proportion of deleterious variants (32 of 381, 8.4%) compared to the random stratified test set (63 of 421, 15.0%), reflecting the uneven clustering of ClinVar-classified pathogenic variants within specific genes and contributing to the lower absolute PR-AUC under gene-stratified evaluation.

Integration of external predictors significantly improved performance across all models under gene-stratified cross-validation. EVE Enhanced, ESM1b Enhanced and AlphaMissense Enhanced each demonstrated statistically significant PR-AUC improvements over their respective standalone predictors. For EVE, the mean cross-validation PR-AUC increased from 0.473 to 0.666 ($p=0.010$), and for ESM1b, the mean PR-AUC increased from 0.600 to 0.710 ($p=0.003$), and for AlphaMissense, the mean PR-AUC increased from 0.703 to 0.753 ($p=0.040$). ROC-AUC improvements followed a similar pattern, with the largest absolute gain observed for EVE (0.706 to 0.856, $p<0.001$) and a significant improvement for ESM1b (0.836 to 0.875, $p=0.017$). The AlphaMissense ROC-AUC improvement did not reach statistical significance (0.897 to 0.905, $p=0.064$), consistent with the limited performance headroom available for the strongest standalone predictor.

Subsequently, on the gene-stratified hold-out test set, AlphaMissense Enhanced and combined Enhanced achieved the highest performance (PR-AUC 0.723 and 0.738; ROC-AUC 0.930 and 0.933, respectively). The combined standalone model without the IDR specific features achieved a gene-stratified test PR-AUC of 0.657 and ROC-AUC of 0.867, lower than all enhanced models, confirming that IDR-specific biophysical features provide complementary discriminative signal even when generalizing to unseen genes. (S6 Table).

Supplementary Figures

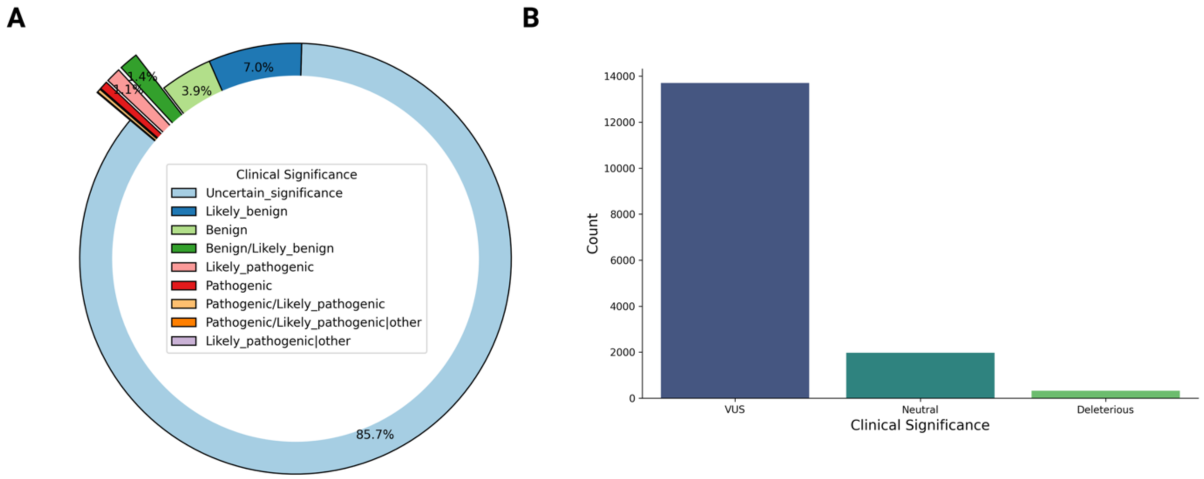

S1 Fig: variant proportions and counts in predicted intrinsically disordered regions (IDRs) in ClinVar. A.) Proportion of variants found in predicted IDRs stratified by clinical significance listed in the ClinVar database. B.) Counts of ClinVar variants after re-grouping the Clinical significance in three functional groups (Deleterious, Neutral and VUS)

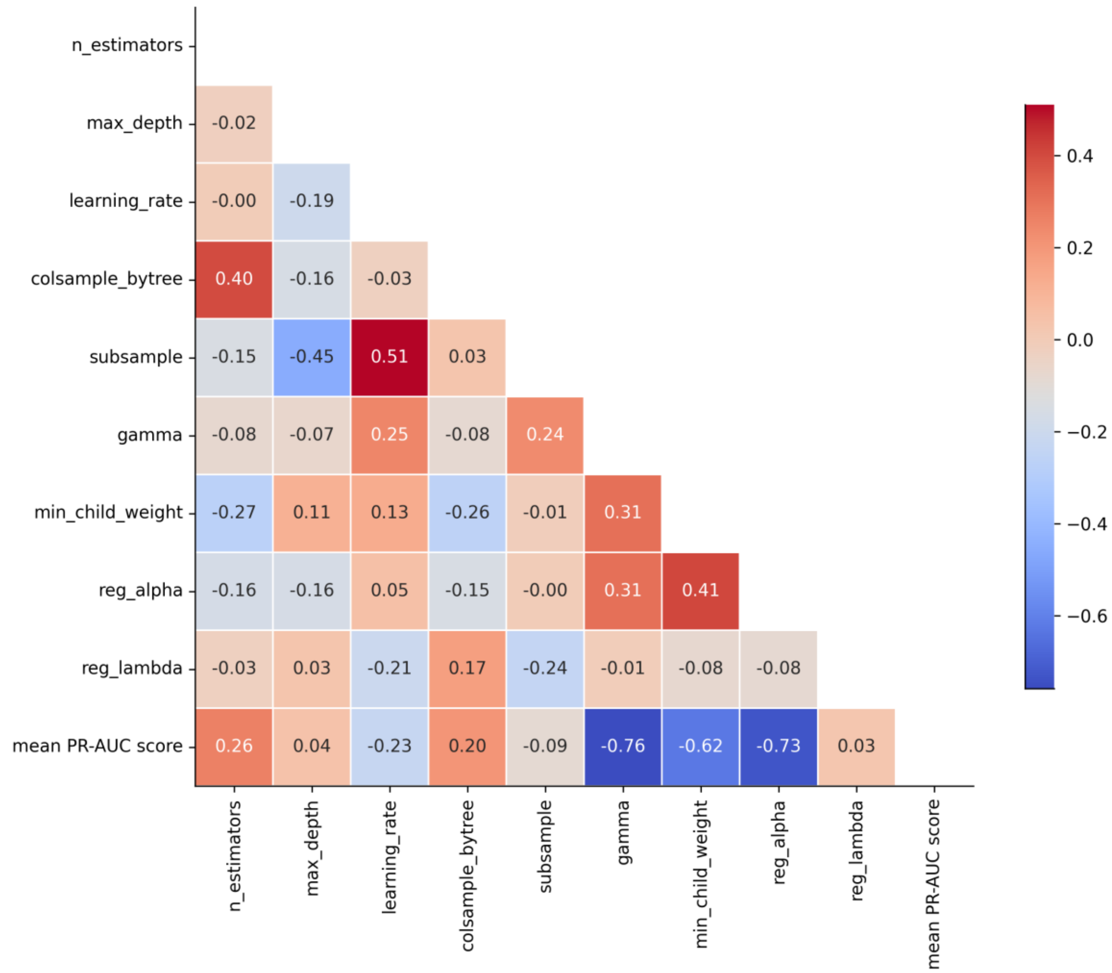

S2 Fig: Correlation of the distribution of hyperparameters setting from XGboost model in predicting protein function from variants in IDRs highlighting the importance of the hyperparameter gamma, min_child_weight and reg_alpha to improvement of mean AUC scores.

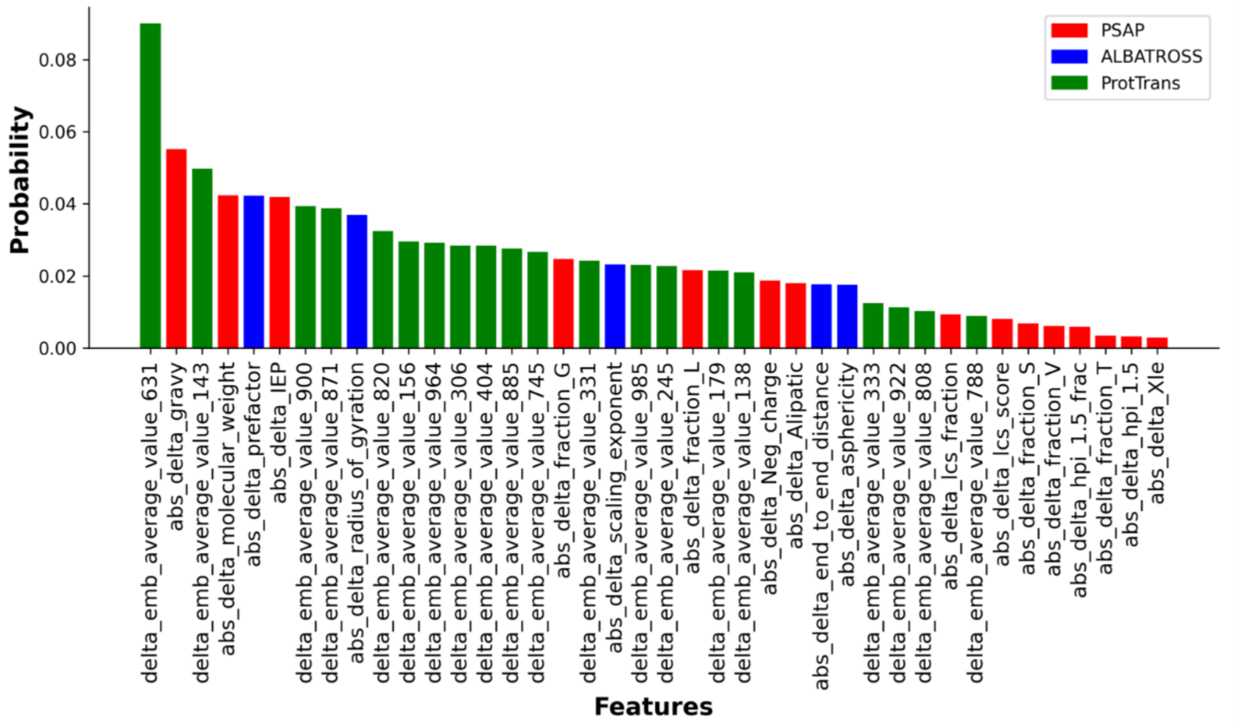

S3 Fig: The top 40 most important feature from the optimized XGboost model trained on features predicting absolute change in phase separation, absolute change in global conformation and average embedding combination in predicting protein function from a missense variant. SHAP probabilities highlights the importance of the features towards model prediction. The colors (red, blue and green) indicate the source of the features.

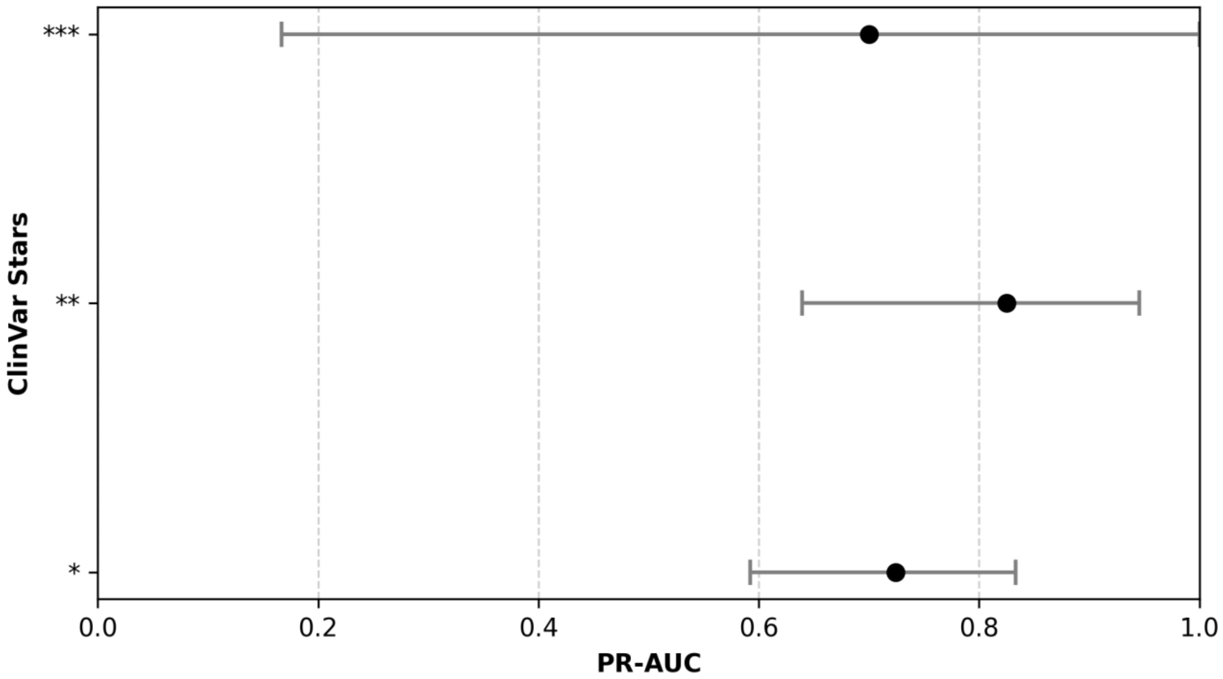

*S4 Fig: Precision-recall area under the ROC curve (PR-AUC) with 95% confidence intervals for the predictive performance of the proposed model on hold-out test variants, stratified by ClinVar review status. Variants are grouped into categories based on ClinVar's star ratings: * for "criteria provided, single submitter", ** for "criteria provided, multiple submitters, no conflicts", and *** for "reviewed by expert panel"*

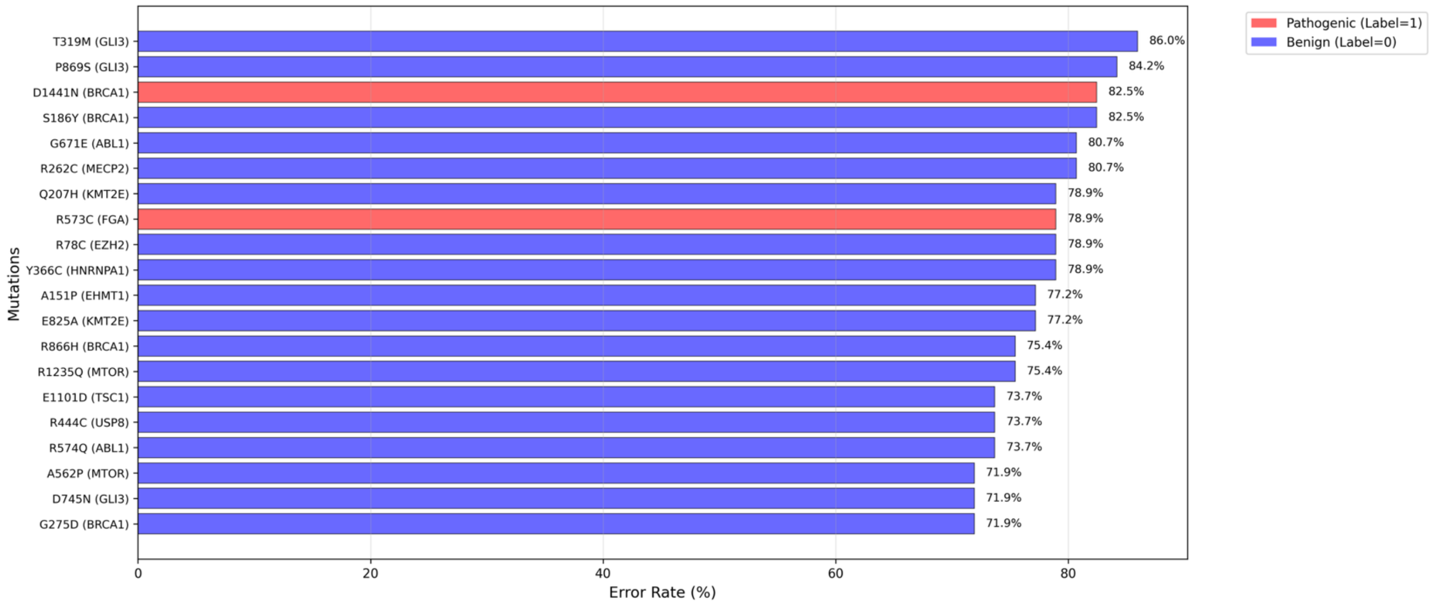

S5 Fig: Comparative error rates for challenging missense variants across in silico predictors. The horizontal bar chart displays error rates (%) for the 20 most difficult-to-classify variants from the 421variant hold-out test dataset, showing

prediction accuracy challenges for both pathogenic (red bars, Label=1) and benign (blue bars, Label=0) classifications. Variants are labeled with their amino acid change and associated gene symbol.

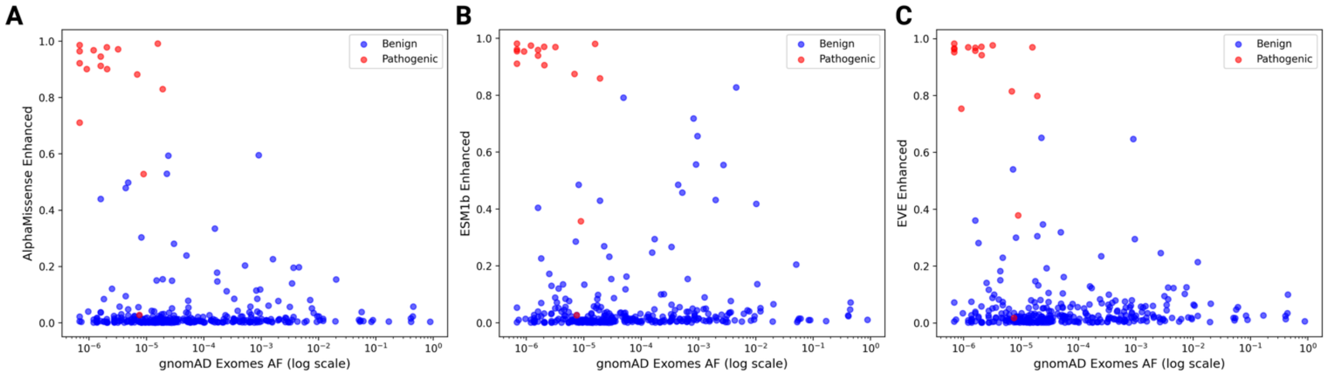

S6 Fig: Association between enhanced model predictions and population allele frequency Relationship between gnomAD Exomes allele frequency (log scale) and pathogenicity predictions from (A) AlphaMissense Enhanced, (B) ESM1b Enhanced, and (C) EVE Enhanced models. Variants are colored by ClinVar classification: pathogenic (red) and benign (blue). Pathogenic variants demonstrate higher prediction scores and lower population frequencies compared to benign variants.
