# Supplementary material for: Enhancing missense variant classification in predicted intrinsically disordered regions
Source: PLoS One. 2026 Jul 27;21(7):e0354365. doi: 10.1371/journal.pone.0354365 (PMC13405113; doi:10.1371/journal.pone.0354365)
Supplement: Attachment — Submitted filename: PLOS_reviewer_response.pdf [file pone.0354365.s004.pdf]

Response to reviewer

Reviewer #1

Comment: I think that there is a need for this form of testing and I congratulate the authors for developing this. It is, however, helpful to have a temporary GUI to test the system using known IRD variants. Without that, it is only useful to tautologically realize that a pathogenic variant is a pathogenic variant

Response: *We thank the reviewer for this suggestion and fully agree that a graphical interface would substantially improve accessibility and usability of the methodology for the broader community. Unfortunately, deployment of a publicly accessible web application is not feasible within our current institutional, and we are unable to obtain the necessary approvals within the revision timeline. However, to address the reviewer's concern about usability, we publish the complete source code, pre-trained model weights, available at our GitHub repository (https://github.com/rohandavidg/IFP-MIDR). We provide a Jupyter notebook that walks through the complete methodology step by step, allowing users with a local Python environment to run predictions on their own variant lists without requiring any institutional infrastructure.*

Reviewer #2

Comment: The manuscript employs distinct processing strategies for different feature types: while ProtTrans embeddings were subjected to four different combination methods (L1, L2, Average, Hadamard), the biophysical features (gIDRc and PS) were processed solely using absolute delta change. It is implied that biophysical features primarily reflect structural stability disruption, where the magnitude of change correlates with pathogenicity. However, since protein embeddings also encode structural and functional information, the rationale for not exploring similar complex interaction terms for biophysical features is unclear. If possible, briefly explaining why similar complex calculations weren't attempted for features (PS and gIDRc) would help readers better understand the innovation of the feature processing strategy.

Response: *We thank the reviewer for raising this methodological question. The distinction in processing strategy between the biophysical features (gIDRc and PS) and the protein language model (pLM) embeddings is deliberate and grounded in the nature of each feature type. The gIDRc and PS features are scalar biophysical quantities (e.g., radius of gyration, end-to-end distance, asphericity, phase separation propensity) derived from sequence-based predictors. Each feature has a well-defined physical interpretation, and the direction of change is not informative in isolation and what matters is the magnitude of the perturbation induced by the variant. Absolute delta change therefore captures the relevant signal. Applying interaction terms such as L1, L2, or Hadamard product to these low-dimensional scalar features would be redundant as L1 and absolute delta are equivalent for scalars, L2 is the Euclidean norm of differences and a Hadamard product of two scalar values yields a cross-feature term that conflates multiple biophysical dimensions without a clear physical rationale. In contrast, pLM embeddings are high-dimensional contextual vectors (2,048 dimensions) where the relative positioning and interaction structure across dimensions encodes complex sequence context. For these, different combination strategies can capture different aspects of the mutational shift in embedding space, as L1 captures axis-aligned differences, L2 captures Euclidean distance, the average summarizes the shared context, and the Hadamard product captures element-wise co-activation patterns. These are meaningfully distinct operations for high-dimensional vectors in a way that does not apply to scalar biophysical quantities. We have now added a clarifying sentence to the Data Preprocessing section of the manuscript.*

Comment: The manuscript demonstrates performance improvements achieved by individually adding prediction scores from AlphaMissense, EVE, and ESM1b as a new feature. To further evaluate the independent contributions and synergistic effects of these external features, would incorporating the following comparative experiments strengthen the evaluation section: (1) assessing model performance using only the scores of these three external predictors (without the authors' proposed features) as input, and (2) evaluating performance when the scores of all three models are all used as input features. This would help clarify whether the improvement is primarily

driven by a single powerful model (such as AlphaMissense) or stems from the complementary fusion of multi features.

Response: *We thank the reviewer for the valuable suggestion. We have now performed both experiments requested and report the results below and have added it into the manuscript*

1. ***External predictors only (AlphaMissense + ESM1b + EVE scores, without IDR specific features):*** *An XGBoost model trained on only the three external predictor scores achieved a hold-out test PR-AUC of 0.8298 and ROC-AUC of 0.9318 demonstrating that these scores alone provide a strong baseline but fall short of our full enhanced models.*

(2) ***All three external scores combined with IDR specific features****: A model integrating AlphaMissense, ESM1b, and EVE simultaneously alongside the IFP-MIDR features achieved a hold-out test PR-AUC of 0.9076 and ROC-AUC of 0.9786.*

These results confirm that the improvement observed in our Enhanced models is not driven solely by AlphaMissense, rather, the IDR specific features provide independent and complementary predictive signal.

Comment: For the "Evaluation of dbNSFP predictors with functions" in the part of Results, where 23 models from the dbNSFP database were evaluated on classifying missense variants, could you specify which dataset of missense variants was used for this evaluation? Was it the test set?

Response: *We thank the reviewer for the question, The dbNSFP evaluation was performed on the full labeled dataset of 2,104 missense variants (316 deleterious, 1,788 neutral) prior to train/test splitting, not on the held-out test set. This was intentional, since the dbNSFP predictors were not trained by us, there is no risk of data leakage from evaluating them on the full dataset, and doing so provides the most stable and representative performance estimates for all 23 tools given the relatively limited sample size. We have added a clarifying sentence to the manuscript "Performance metrics for all 23 dbNSFP predictors were computed on the full labeled dataset of 2,104 variants, as these models were not trained by the authors and are therefore not subject to overfitting concerns with respect to our data split."*

Comment: The figures in the manuscript have low clarity, making them difficult to read. If all figures could be replaced with high-clarity vector graphics (such as EPS, or SVG formats), it would allow data details to be clearly displayed during reading.

Response: *We thank the reviewer for the suggestion, we have now updated the figures in the manuscript to improve the clarity.*

Comment: Currently, the description of feature processing in the manuscript structure is relatively fragmented: the delta change processing for PS and gIDRc is introduced in the "Model generation" section, while the four computational methods for embedding are detailed in the "Data preprocessing" section. It would enhance the coherence of the article if all content of Feature preprocessing (including the delta change for physical features and the four computational strategies for embedding) could be consolidated into a single section for a unified introduction. Alternatively, briefly previewing the processing workflow for embedding in the "Model generation" section would also improve readability.

Response: *We thank the reviewer for this suggestion. We agree that the fragmented presentation of feature processing impedes readability. In the revised manuscript, we have consolidated all feature preprocessing content into a single dedicated subsection titled "Feature preprocessing" within the Materials and Methods. This unified section now covers: (1) the absolute delta change computation for gIDRc and PS biophysical features, (2) the four embedding combination strategies (L1, L2, Average, Hadamard) for ProtTrans embeddings, and (3) the rationale for the distinct treatment of each feature type. We believe this reorganization meaningfully improves the logical flow of the Methods.*

Comment: Figure 1C illustrates the processing workflow for WT and Mutant embeddings. If I understand correctly, the current figure may not fully reflect the compression process from "sequence embeddings matrix to single vector." If convenient to modify, it is suggested to more intuitively display this embedding of sequence change in the figure: explicitly label the embeddings as an L×2048 matrix (L is the amino acid sequence length), which becomes a 1×2048 vector after Average Pooling. This will help readers understand the features embeddings transformation process more quickly.

Response: *We thank the reviewer for this helpful observation. We have revised Figure 1C to explicitly label the ProtTrans output as an L×1024matrix (where L is the amino acid sequence length of the IDR) and to show the global average pooling step that reduces this to a 1×2048 vector, as well as showing the final 20 embedding dimensions used as features. The revised figure also labels each step and the total features extracted.*

Reviewer #3

Comment: The manuscript is not clearly written and words are used inconsistently. Readers need to know in advance what kinds of information were included in each dataset or generated by each tool. Clearly state which features in which dataset or tool were used, and how these features were used in the analysis. The Feature Generation section starting from line 190 may include the required information. Reorganize the manuscript.

Response: *We thank the reviewer for the suggestion. We have substantially revised the manuscript to improve clarity and consistency. Terminology is now standardized throughout, "neutral" replaces "benign", "ROC-AUC" replaces "AUC", and embedding dimensionality is corrected from 2048 to 1024. The Materials and Methods has been reorganized into four clearly delineated sections: Feature Generation (describing what each tool produces, 5 gIDRc features from ALBATROSS, 48 PS features from BioPython, and a 1024-dimensional embedding per sequence from ProtTransBertBFD), Feature Preprocessing (describing how WT and mutant values are combined into variant-level absolute delta features, how the four embedding combination strategies are applied, and how F-statistic feature selection and standardization are performed within cross-validation folds), Model Creation (describing classifier comparison and cross-validation strategies), and Hyperparameter Optimization (describing the Optuna framework and gene-stratified splitting). Redundant sections have been removed and Figure 1C has been updated to explicitly annotate each step with its input and output dimensionality.*

Comment: Abstract. Words representing feature names used in the abstract are different from those in the manuscript. Use consistent words throughout the manuscript

Response: *We thank the reviewer for highlighting this inconsistency, the abstract has been revised to use terminology that is consistent with the Materials and Methods throughout. Specifically: "features predicting IDR properties that infer global IDR conformation" has been replaced with "global IDR conformation (gIDRc) features from ALBATROSS"; "features predicting phase separation" has been replaced with "phase separation (PS) features from BioPython"; "protein embeddings derived from a protein language model" has been replaced with "1024-dimensional protein embeddings from ProtTransBertBFD"*

Comment: Introduction. Reviewer does not understand what authors hope to indicate by showing variants in HBB and FGFR3. The phenotypic difference arises from the difference in the variant genes. These examples have no relevance to the current study.

Response: *We agree and we have removed these examples from the Introduction and replaced them with examples of pathogenic missense variants documented to occur in IDRs, which are more directly germane to the study's rationale. We have now added an example from TP53 in the*

introduction "For example, the K24N missense variant in TP53 reduces binding affinity to key regulatory partners, compromising p53-mediated tumor suppression"

Comment: Introduction. References 6 to 11 are insufficient. There are more than 30 similar models. At least commonly used models should be included in the references.

Response: *We have now expanded the Introduction to include a broader set of computational missense predictors beyond the original citations of SIFT, PolyPhen-2, BayesDel, and CADD. The revised Introduction now cites meta-predictors, including REVEL, ClinPred, and VEST4, which aggregate scores from multiple individual tools, as well as PrimateAI and MutPred2, which represent distinct methodological approaches: PrimateAI leverages evolutionary constraint from non-human primate variation, while MutPred2 incorporates the 3D structure and free energy to predict the molecular mechanisms of pathogenicity.*

Comment: Introduction. "However, a large portion of missense variants remain unclassified due to the rarity in the population." This statement is confusing. Rarity is not the cause of uncertainty.

Response: *We thank the reviewer for the comment and agree, we have now revised this sentence to "However, a large proportion of missense variants remain classified as variants of uncertain significance (VUS), in part because many occur at low population frequencies that preclude robust statistical evidence for or against pathogenicity, and because functional characterization of individual variants at scale remains impractical."*

Comment: Figure 1 shows that the ALBATROSS score, the IDR-relevant compositional features, and the ProtTrans score were used to make a binary model to classify disease-causing IDR variants. This scheme is against what are explained in the abstract or the manuscript. Figure 1C. Biopython is not addressed in the manuscript or the legend. Figure 1C. "Average" of ProtTrans is difficult to understand. This is also applicable to the Methods. Why was averaging able to show the difference between the wild-type and mutant IDRs?

Response: *We thank the reviewer for raising this important observation. Figure 1C has been substantially revised to address all three points. Regarding the discrepancy between Figure 1C and the manuscript description, we agree with the reviewed and Figure 1C has been redrawn to clearly show that the absolute delta values (|mutant – Wild Type (WT)|) of the gIDRc and PS features are computed prior to model input, and that it is these variant-level perturbation features and not the raw WT or mutant scores, that enter the final feature table. The pipeline now explicitly shows WT and mutant sequences processed in parallel, with the delta computation as a distinct intermediate step.*

BioPython is now explicitly labelled in Figure 1C and described in the Methods. BioPython is used via the "ProteinAnalysis" class to compute 48 phase separation compositional features per sequence. These features are computed independently for the WT and mutant IDR sequences, and absolute delta values are taken for each.

Regarding the "Average" label for ProtTrans, we acknowledge this was ambiguous because it conflated two distinct averaging operations. Figure 1C now explicitly annotates both steps with their input and output dimensionalities. The first operation is mean pooling across residue positions. For a sequence of length L, ProtTransBertBFD generates an L × 1024 per-residue embedding matrix, and the mean across all L positions yields a single 1024-dimensional sequence-level vector, performed separately for the WT and mutant sequences. The second operation is the WT-mutant combination strategy, the element-wise average (WT + mutant) / 2 of the two 1024-dimensional vectors produces a single variant-level representation encoding the shared IDR sequence context. We evaluated four such combination strategies (L1, L2, Average, Hadamard) and the Average combination achieved the highest cross-validation PR-AUC. We hypothesize that for IDR missense variants, where single amino acid substitutions cause small perturbations to a flexible sequence, the shared contextual representation captures the functional identity of the IDR more robustly than the difference alone. This is now explained explicitly in the revised Methods and Figure 1C legend.

Comment: Model generation and other sections. How were IDRs selected? Authors repeatedly state that IDRs were selected by AlphaFold-RSA, but they also state that IDRs are taken from MobiDB. If AlphaFold-RSA was used, indicate their thresholds or categories. If MobiDB was used, which categories were recognized as IDRs. Give a rationale why the threshold of 0.581 was chosen. Also explain what this score is.

Response: *We thank the reviewer for the question. We agree that the original manuscript did not provide clarity and we have updated this. We have updated the "model generation" section in the manuscript to provide more clarity. IDR coordinates were obtained by downloading the prediction-disorder-AlphaFold annotation from MobiDB. Within MobiDB, the prediction-disorder-AlphaFold annotation is computed using two AlphaFold2-derived definitions of disorder. The first is based on the pLDDT score, where residues with pLDDT lower than 70% are considered disordered. The second definition uses per-residue relative solvent accessibility (RSA) computed by DSSP, averaged over a sliding window of 25 residues, with positions having average RSA above 0.58 classified as disordered. Residue annotations are calculated using the AlphaFold-disorder script applied to AlphaFold Protein Structure Database predictions. The contiguous IDR segments defined by these coordinates were extracted and used to identify missense variants from ClinVar whose protein position fell within a predicted IDR interval. These mutations were used in the analysis*

Comment: Line 177. Why the variants were largely reduced from 15,999 to 2,203. If VUSs were excluded indicate as such. ClinVar variants were mapped to dbNSFP. The statement is opposite. Explain why the number of variants was reduced from 2203 to 2104. dbNSFP is a database of saturation mutagenesis and should include all available missense variants.

Response: *We thank the reviewer for their comment and have now explicitly mention that the reduction from 15,999 to 2,203 variants, and from 2,203 to 2,104 based on two sequential filters: (1) exclusion of VUS-classified variants (which constitute 85.7% of all IDR ClinVar variants), retaining only pathogenic/likely pathogenic (deleterious) and benign/likely benign (neutral) variants; and (2) restriction to genes with RefSeq transcripts listed in ClinVar that could be unambiguously mapped to an orthologous Ensemble transcript listed in dbNSFP v4.8. It is this reason why the mutation count drop from 2,203 to 2,104. Regarding the mapping statement, we agree with the reviewer and have updated the manuscript to now state that "Variants from the filtered ClinVar IDR dataset were intersected with dbNSFP v4.8 to retrieve available in silico prediction scores for each variant."*

Comment: Line 200. Reviewer supposes that "2048-dimensional embeddings for both mutant and WT sequences for every amino acid" should be "2048-dimensional embeddings for every amino acid in both mutant and WT sequences". This kind of incorrect statements make this manuscript difficult to understand. Line 201. How were the embeddings averaged? The dimensions were reduced from 2048 to 1 for each amino acid or the 2048 dimensions remained unchanged but each score was averaged along the entire amino acids. Which is correct?

Response: *We thank the reviewer for this correction. The original sentence was grammatically ambiguous and has been revised. The correct interpretation is that ProtTransBertBFD generates a 1024-dimensional embedding vector for every amino acid position in the input sequence, producing an $L \times 1024$ matrix for a sequence of length L. This is performed separately for the WT and mutant IDR sequences. We have corrected the sentence to read: "ProtTransBertBFD generates 1024-dimensional embeddings for every amino acid in both the WT and mutant IDR sequences."*

The averaging operates across amino acid positions, not across embedding dimensions. Specifically, for a sequence of length L, ProtTransBertBFD produces an $L \times 1024$ matrix where each row is the 1024-dimensional embedding of one amino acid. The mean is then computed across all L rows (amino acid positions), yielding a single 1024-dimensional vector that represents the entire sequence. The 1024 embedding dimensions are preserved throughout, it is the sequence length dimension that is reduced from L to 1. This operation is known as mean pooling and is standard practice for obtaining fixed-length sequence-level representations from transformer-based protein language models. This process is performed independently for the WT and mutant sequences, producing one 1024-dimensional vector per sequence. We have rewritten the relevant

Methods sentence in feature generation section to make this explicit: "To incorporate sequence-level representations, we used the pLM ProtTransBertBFD to generate per-residue embeddings for both WT and mutant IDR sequences. For a sequence of length L, ProtTransBertBFD produces an L × 1024 embedding matrix, where each row corresponds to the 1024-dimensional representation of one amino acid position. Mean pooling was then applied across all L residue positions to yield a single 1024-dimensional sequence-level vector per sequence, a standard approach for obtaining fixed-length representations from transformer-based protein language models. This process was performed independently for the WT and mutant sequences, producing one 1024-dimensional vector for each."

Comment: Line 204. The process to reduce the dimension to 20 is not clearly stated. In line 201, authors state 1 single embedding. In addition, feature elimination process always causes overfitting. Explain in detail how the features were eliminated and show that testing dataset was not touched in the process of feature elimination.

Response: *We thank the reviewer for this methodological comment. In our method, following mean pooling, each variant is represented by two 1024-dimensional vectors, one for the WT sequence and one for the mutant sequence. These are combined into a single variant-level representation using one of four combination strategies (L1, L2, Average, Hadamard), each producing a 1024-dimensional vector per variant. It is this 1024-dimensional combined embedding vector that is then subject to dimensionality reduction. F-statistic feature selection is applied to identify the 20 embedding dimensions most associated with the binary pathogenicity label across the training data. The remaining 53 biophysical features (5 gIDRc and 48 PS absolute delta features) are low-dimensional scalar quantities that are retained in full and are not subject to any dimensionality reduction. The final feature matrix per variant therefore comprises 20 selected embedding dimensions plus 53 biophysical features, giving 73 features in total.*

We confirm that in the revised analysis, feature selection is performed exclusively on training data, and the test dataset is never accessed during this process. Specifically, within each cross-validation fold, the F-statistic selector is fitted using only the training fold variants and the resulting selected indices are then applied to transform the held-out validation fold. The selector is never exposed to validation or test data. Similarly, in the hyperparameter optimization stage, the dataset is first divided into an 80% training partition and a 20% held-out test set. Feature selection is fitted on the 80% training partition only, and the identical selected indices are applied to transform the held-out 20% test set prior to evaluation. The test set is not used at any point during feature selection, model training, or hyperparameter optimization. We have rewritten the "Feature Preprocessing" section to make this procedure explicit.

Comment: Lines 246, 257, 290 and others. AUC of what? ROC-AUC, PR-AUC, or something else?

Response: *This has been corrected in the updated manuscript; we have replaced every instance of "AUC" with explicit designation of "ROC-AUC" or "PR-AUC" as appropriate*

Comment: Legend for Figure 3A. Reviewer does not understand how and where square root was applied. In line 331, square root was applied to four features. How about Scaling Exponent?

Response: *We thank the reviewer for this observation. The square root and log transformations were applied exclusively for visualization purposes when generating the Figure 3A boxplots and were not applied at any point in the feature generation, model training, cross-validation, or hyperparameter optimization pipeline. The untransformed absolute delta values ($|WT - mutant|$) for all five gIDRc features are used as model inputs throughout. for Figure 3A, four features asphericity, radius of gyration, end-to-end distance, and prefactor were square root transformed. The scaling exponent was log transformed rather than square root transformed, because its distribution is more heavily right-skewed and the log transformation provided better visual separation between the Deleterious and Neutral classes. We have updated the Figure 3A legend to state explicitly for each feature which transformation was applied for display purposes and have added a note clarifying that these transformations are for visualization only and do not affect any reported statistical results or model inputs.*

Comment: Legend for Figure 3B. AUC is not indicated in the figure. ROC is shown in the figure.

Response: *The legend has been revised to: "(A) Association of the absolute change in Radius of Gyration, Asphericity, Scaling Exponent, Prefactor, End to End distance between WT and mutant IDR sequences, stratified by ClinVar pathogenicity classification (deleterious in red, neutral in blue). Square root transformation was applied to radius of gyration, asphericity, prefactor, and end-to-end distance for visualization. Log transformation was applied to scaling exponent. All statistical comparisons were performed on untransformed values using the Mann-Whitney U test. (B) ROC-AUC curves for each individual gIDRc feature. (C) Precision-recall curves for each individual gIDRc feature. The random baseline PR-AUC of 0.14 reflects the positive class prevalence in the dataset."*

Comment: Line 346. Reviewer supposes that the four combination methods were applied to embeddings of ProtTrans. Clarify this. Otherwise, the story is difficult to follow. In addition, gIDRc and PS used in Figure 4 are not always used in other figures or in the manuscript. This discrepancy causes confusion.

Response: *We thank the reviewer for the comment. The four embedding combination strategies (L1, L2, average, Hadamard) were applied exclusively to the ProtTrans pLM embeddings; the gIDRc and PS biophysical features were processed using absolute delta change throughout all experiments. We have now added verbiage in the "Feature preprocessing" section to clarify this. We have also standardized gIDRC and PS abbreviations across all figures, legends, and the main text. Both abbreviations are now introduced at first use with full definitions and are used consistently in all subsequent figures. Figure 4 legends have been updated to explicitly state that all four embedding combination models include gIDRc and PS features in addition to the pLM embeddings.*

Comment: Legend for Figure 4A. "Accuracy performance" is inaccurate. Authors are showing ROC-AUC. Legend for Figure 4B. Clarify that PR-AUC is indicated.

Response: *The legends of Figure 4 has been corrected in the updated manuscript. It now states* **"***Performance comparison of multiple protein embedding combination methods using multiple machine learning models to predict protein function on classified variants found in ClinVar in predicted IDR regions. (A) ROC-AUC and* ***(B)*** *PR-AUC distributions across 10-fold cross-validation for four embedding combination methods (L1, L2, Hadamard, Average) combined with gIDRc and PS features, evaluated using Random Forest, MLP, and Naïve Bayes classifiers. Significance brackets indicate pairwise comparisons with Bonferroni-corrected p-values from Dunn's post hoc test following Kruskal-Wallis testing (* p<0.05, ** p<0.01, *** p<0.001)".*

Comment: Figure 4. Show statistical significance by two-way ANOVA or Kruskal-Wallis test with an appropriate post hoc test.

Response: *We have added appropriate statistical testing to Figure 4. Given that the cross-validation fold-level metric distributions cannot be assumed to follow a normal distribution, we used the Kruskal-Wallis test to assess overall differences across embedding combination methods for each classifier, followed by pairwise Mann-Whitney U tests with Bonferroni correction as a post hoc procedure. Significant pairwise comparisons are now annotated on the figure with standard notation (ns, *, **, ***).*

Comment: Line 376. Show the effect of HPO on ROC-AUC. PR-AUC is not sufficient.

Response*: We thank the reviewer for the suggestion. ROC-AUC is now reported alongside PR-AUC for all experiments, including the hyperparameter optimization results, the cross-validation embedding comparison (Figure 4), and the hold-out test set evaluation (Table X). We note that hyperparameter optimization was performed by maximizing PR-AUC rather than ROC-AUC as the primary objective, because the dataset is substantially imbalanced (322 deleterious variants versus 1,973 neutral variants, positive rate 14%). Under class imbalance, ROC-AUC can be misleadingly optimistic, a classifier that assigns high scores to the majority class can achieve high ROC-AUC while failing to identify pathogenic variants at clinically relevant precision levels. PR-AUC directly measures performance on the minority (deleterious) class and is therefore the more appropriate optimization target for this application. Nevertheless, we agree that reporting ROC-AUC alongside PR-AUC provides a more complete picture of model performance, and both metrics are now reported consistently throughout the revised manuscript*.

Comment: Line 380. Leakage by checking exact sequences should not be included in Results. Authors do not even have to address this in Materials and Methods, although this should be carefully scrutinized when making a dataset.

Response: *We thank the reviewer for their comment and we agree. The sentence describing pairwise sequence identity checks has been removed from the Results section. The check was performed during dataset construction and is now briefly mentioned in the Data Selection subsection of Methods as part of the data quality assurance procedure, without occupying space in the Results.*

Comment: S3 Fig. What is the last number 631 in the best feature in the SHAP score? Lack of clear explanation in the method prevents the reviewer understand what is presented in this figure.

Response: *We thank the reviewer for identifying this lack of clarity. The number 631 refers to the index of a specific dimension within the 1024-dimensional ProtTransBertBFD embedding space. As described in the Methods, F-statistic feature selection (SelectKBest, ANOVA F-test) is applied to reduce the 1024-dimensional combined embedding vector to the 20 most discriminative dimensions. Each selected dimension retains its original index from the full 1024-dimensional space to ensure traceability, dimension 631 was ranked highest by SHAP importance among the 20 selected embedding dimensions, indicating it contributes most strongly to model predictions. This does not imply that dimensions 0–630 were all retained, rather, 20 dimensions were selected from across the full 1024-dimensional space, and dimension 631 happened to be the most informative of those selected. The Figure S3 legend and Methods have been revised to make this explicit.*

Comment: Baseline is shown three times and vertical scales are different in three panels. Make a single graph with baseline, EVE alone, ESM1b alone, AlphaMissense alone, EVE enhanced, ESM1b enhanced, and AlphaMissense enhanced, so that the performances of these models can be easily recognized. Legend for Fig. 5. Multiple comparisons are not adjusted for

Response: *Thank you for your comment, we have updated fig 5 to show the PR-AUC and ROC-AUC of the standalone vs enhanced models. We have updated the legend to highlight that no multiple testing was done*.

Comment: *S4 Fig. Show statistical significance.*

Response: *We thank the reviewer for this suggestion. Formal statistical comparison between ClinVar review status categories was not performed for Figure S4 for the following reasons. The three-star categories contain highly unequal and small sample sizes. The three-star (expert panel) category contains only a small number of variants, as expert panel-reviewed IDR variants are rare in ClinVar. With such small and unequal group sizes, any statistical test comparing PR-AUC estimates across star categories would have insufficient power to detect meaningful differences, and the resulting p-values would be unreliable. Furthermore, the confidence intervals shown in Figure S4 are already wide for the lower star categories, visually conveying the uncertainty in these estimates.*

Comment: Section starting from line 463 and S5 Fig. Showing only favorable variants is not fair and misleading. Remove this section. If authors hope to keep this section, show variants that resulted in unfavorable prediction outcomes in the authors' models.

Response: *We thank the reviewer for this comment. The variants shown in Figure S5 were not preselected to favor our model. Rather, these are the top 20 variants with the highest misclassification rate across all 56 dbNSFP in silico predictors, that is, variants that the existing computational tools collectively struggle to classify correctly, regardless of our model's performance on them. The selection criterion is therefore based entirely on the error rate of existing predictors, not on the performance of our method. We then assessed how our methodology performed on this challenging subset, which includes cases where our model also fails. The figure legend and the corresponding Methods section have been revised to make this selection criterion explicit: "The top 20 variants are ranked by their misclassification rate across all 56 dbNSFP predictors evaluated in this study. Variants are colored by their true ClinVar classification"*

Comment: Authors are showing that the combination of their tool with either AlphaMissense, ESM1b, or EVE markedly improved the performance. Why were all the three previously reported scores not added the authors' model? Reviewer also hope to know whether the combination of the three previous scores outperformed the authors' model alone.

Response: *We thank the reviewer for this suggestion and we agree. We have now trained and evaluated a model incorporating all three external predictors simultaneously alongside our method derived features. Additionally, we evaluated a combined standalone model using all three predictor rankscores without the gIDRC, PS, and PLM features (AlphaMissense + ESM1b + EVE standalone), to directly address the question of whether combining the three existing tools alone outperforms the enhanced model, the combined standalone model (AlphaMissense + ESM1b + EVE) achieved PR-AUC 0.761 ± 0.031 and ROC-AUC 0.903 ± 0.032. Adding features predictive of gIDRC, PS, and pLM to all three predictors simultaneously improved PR-AUC to 0.864 ± 0.035 (+0.104, p<0.001) and ROC-AUC to 0.944 ± 0.032 (+0.041, p=0.007), both statistically significant. These results are now reported in the revised Results section and Figure 5.*

Comment: Section starting from line 472. A statement "EVE Enhanced showing the most pronounced bimodal distribution" cannot be recognized in S6 Fig or anywhere else. Variants with high minor allelic frequency (MAF) can be easily recognized as benign variants. We do not have to use any tool for such variants. Excluding these variants would make a balanced dataset with similar numbers of benign and mutant variants. Reviewer suggests making models after excluding these high MAF variants.

Response: *We thank the reviewer for the observations. Regarding the bimodal distribution statement, we agree this claim is not clearly supported by Figure S6 and have removed it from the revised manuscript. The text now states more precisely that all three enhanced models assign high*

prediction scores to the majority of true pathogenic variants and low scores to the majority of benign variants, with the clearest separation observed at low gnomAD allele frequencies. Regarding high MAF variants, the overwhelming majority of variants in our dataset are extremely rare, most benign variants cluster at gnomAD Exomes AF below 10^{-4}*, reflecting the nature of ClinVar curated IDR variants which are predominantly rare clinical findings rather than common population polymorphisms.*
