# Supplementary material for: Enhancing missense variant classification in predicted intrinsically disordered regions
Source: PLoS One. 2026 Jul 27;21(7):e0354365. doi: 10.1371/journal.pone.0354365 (PMC13405113; doi:10.1371/journal.pone.0354365)
Supplement: Attachment — Submitted filename: PLOS_ONE.ResponsetoReviewer3.docx [file pone.0354365.s005.docx]

**Response to Reviewer 3**

We thank the reviewer for their comments on the manuscript. We have addressed all four comments as detailed below.

**Comment 1:** Line 51. Line 51. A statement on K24N in TP53 still does not fit to this section. Even if K24N is in IDR, the relevance of IDR has not been addressed up to this point. In addition, a reference is missing for this statement. Eliminate this statement. Alternatively, show an example of two different amino acid substitutions at an identical amino acid resulted in mild and severe phenotypes.

**Response:** *The TP53 K24N example was premature at this point in the Introduction, as intrinsically disordered regions had not yet been introduced. The statement has been removed. In its place, we have substituted it with “For example, the Glu6Val substitution causes sickle cell disease through polymerization of deoxygenated hemoglobin, while the Glu6Lys substitution results in hemoglobin C disease, a clinically milder condition characterized by hemolytic anemia without the vaso-occlusive complications characteristic of sickle cell disease”, which illustrates how different amino acid substitutions at the same position can produce markedly different clinical severities. A reference has been added for this example.*

**Comment 2:** Line 55. “However, a large proportion of missense variants remain classified as VUS, in part because many occur at low population frequencies that preclude robust statistical evidence for or against pathogenicity.” Low MAF is “in favor of” pathogenicity and should not be “against” pathogenicity. The reviewer supposes that the authors hope to state that low MAF alone is not sufficient to conclude that VUS is pathogenic.

**Response:** *The original sentence was misleading. It has been revised to, "However, a large proportion of missense variants remain classified as variants of uncertain significance (VUS), in part because many occur at low population frequencies that, while consistent with pathogenicity under purifying selection, are insufficient alone to establish a causal role in disease, and because functional characterization of individual variants at scale remains impractical." This accurately reflects that low allele frequency is consistent with pathogenicity but not sufficient to confirm it.*

**Comment 3:** Line 56. Spell out VUS in its first occurrence.

**Response:** *VUS is now spelled out as "variants of uncertain significance (VUS)" at its first occurrence in the revised manuscript.*

**Comment 4:** Line 61. “These models employ machine and deep learning techniques trained on common variants observed in non-human primates as a proxy.” Most models have been trained on variants in humans and not in non-human primates. Some models take evolutional information, but most are not restricted non-human primates. If authors are confident, clearly indicate which models were based on non-human primates and which were on humans.

**Response:** *The original sentence incorrectly implied that most models are trained* ***exclusively*** *on non-human primate variants. The sentence has been revised to accurately distinguish training approaches. We updated the introduction with the following:*

*Many of these models are trained on human variant datasets, including population databases and clinical repositories such as ClinVar and gnomAD. A subset leverage evolutionary signals derived from cross-species sequence alignments, while PrimateAI specifically uses common variants observed in non-human primates as a proxy for benign variation, under the assumption that variants tolerated across primate species are unlikely to be pathogenic in humans*
