# Supplementary material for: Enhancing missense variant classification in predicted intrinsically disordered regions
Source: PLoS One. 2026 Jul 27;21(7):e0354365. doi: 10.1371/journal.pone.0354365 (PMC13405113; doi:10.1371/journal.pone.0354365)
Supplement: Peer review history

# Peer review: Decision Letter 0

**DOI:** 10.1371/journal.pone.0354365.r001

**Authors:** Tomoyoshi Komiyama

16 Mar 2026

PONE-D-26-04061

Enhancing Missense Variant Classification in Predicted Intrinsically Disordered Regions.

PLOS One

Dear Dr. Hart,

Thank you for submitting your manuscript to PLOS ONE. After careful consideration, we feel that it has merit but does not fully meet PLOS ONE’s publication criteria as it currently stands. Therefore, we invite you to submit a revised version of the manuscript that addresses the points raised during the review process.

Please submit your revised manuscript by Apr 30 2026 11:59PM. If you will need more time than this to complete your revisions, please reply to this message or contact the journal office at plosone@plos.org. When you're ready to submit your revision, log on to https://www.editorialmanager.com/pone/ and select the 'Submissions Needing Revision' folder to locate your manuscript file.

Please include the following items when submitting your revised manuscript:

- A letter that responds to each point raised by the academic editor and reviewer(s). You should upload this letter as a separate file labeled 'Response to Reviewers'.
- A marked-up copy of your manuscript that highlights changes made to the original version. You should upload this as a separate file labeled 'Revised Manuscript with Track Changes'.
- An unmarked version of your revised paper without tracked changes. You should upload this as a separate file labeled 'Manuscript'.

If you would like to make changes to your financial disclosure, please include your updated statement in your cover letter. Guidelines for resubmitting your figure files are available below the reviewer comments at the end of this letter.

If applicable, we recommend that you deposit your laboratory protocols in protocols.io to enhance the reproducibility of your results. Protocols.io assigns your protocol its own identifier (DOI) so that it can be cited independently in the future. For instructions see: https://journals.plos.org/plosone/s/submission-guidelines#loc-laboratory-protocols. Additionally, PLOS ONE offers an option for publishing peer-reviewed Lab Protocol articles, which describe protocols hosted on protocols.io. Read more information on sharing protocols at https://plos.org/protocols?utm_medium=editorial-email&utm_source=authorletters&utm_campaign=protocols.

We look forward to receiving your revised manuscript.

Kind regards,

Tomoyoshi Komiyama, Ph.D

Academic Editor

PLOS One

**Journal Requirements:**

1. When submitting your revision, we need you to address these additional requirements.

Please ensure that your manuscript meets PLOS ONE's style requirements, including those for file naming. The PLOS ONE style templates can be found at

https://journals.plos.org/plosone/s/file?id=wjVg/PLOSOne_formatting_sample_main_body.pdf and

https://journals.plos.org/plosone/s/file?id=ba62/PLOSOne_formatting_sample_title_authors_affiliations.pdf

2. Please upload a new copy of Figures 2, S1, S5 and S6, as the detail is not clear. Please follow the link for more information:  https://journals.plos.org/plosone/s/figures

3. Please include captions for your Supporting Information files at the end of your manuscript, and update any in-text citations to match accordingly. Please see our Supporting Information guidelines for more information: http://journals.plos.org/plosone/s/supporting-information.

4. If the reviewer comments include a recommendation to cite specific previously published works, please review and evaluate these publications to determine whether they are relevant and should be cited. There is no requirement to cite these works unless the editor has indicated otherwise.

**Additional Editor Comments:**

Dear authors,

Although prediction models have advanced, accurate classification of missense variants in intrinsically disordered regions (IDRs) remains challenging.

By integrating IDR-specific conformational features, phase separation propensity, and protein embeddings, your framework aims to improve prediction accuracy.

However, I think that it is necessary to strengthen the reliability of these results by adding as much information as possible.

We thus have some questions and suggestions for the manuscript that you might consider.

I believe these comments will be helpful in the revision of your study.

Tomoyoshi Komiyama

[Note: HTML markup is below. Please do not edit.]

Reviewers' comments:

Reviewer's Responses to Questions

**Comments to the Author**

1. Is the manuscript technically sound, and do the data support the conclusions?

The manuscript must describe a technically sound piece of scientific research with data that supports the conclusions. Experiments must have been conducted rigorously, with appropriate controls, replication, and sample sizes. The conclusions must be drawn appropriately based on the data presented.

Reviewer #1: Partly

Reviewer #2: Yes

Reviewer #3: Partly

**********

2. Has the statistical analysis been performed appropriately and rigorously?

Reviewer #1: Yes

Reviewer #2: Yes

Reviewer #3: No

**********

3. Have the authors made all data underlying the findings in their manuscript fully available?

The PLOS Data policy requires authors to make all data underlying the findings described in their manuscript fully available without restriction, with rare exception (please refer to the Data Availability Statement in the manuscript PDF file). The data should be provided as part of the manuscript or its supporting information, or deposited to a public repository. For example, in addition to summary statistics, the data points behind means, medians and variance measures should be available. If there are restrictions on publicly sharing data—e.g. participant privacy or use of data from a third party—those must be specified.

Reviewer #1: No

Reviewer #2: Yes

Reviewer #3: Yes

**********

4. Is the manuscript presented in an intelligible fashion and written in standard English?

PLOS ONE does not copyedit accepted manuscripts, so the language in submitted articles must be clear, correct, and unambiguous. Any typographical or grammatical errors should be corrected at revision, so please note any specific errors here.

Reviewer #1: Yes

Reviewer #2: Yes

Reviewer #3: No

**********

5. Review Comments to the Author

Please use the space provided to explain your answers to the questions above. You may also include additional comments for the author, including concerns about dual publication, research ethics, or publication ethics. (Please upload your review as an attachment if it exceeds 20,000 characters)

**Reviewer #1:**  I think that there is a need for this form of testing and I congratulate the authors for developing this. It is however, helpful to have a temporary GUI to test the system using known IRD variants. Without that, it is only useful to tautologically realize that a pathogenic variant is a pathogenic variant.

**Reviewer #2:**  The manuscript is technically sound and the data support the conclusions.

The statistical analysis appears to have been performed appropriately.

The data underlying the findings have been made available.

The manuscript is presented in an intelligible fashion.

1.Summary of the Manuscript

This manuscript presents IFP-MIDR, a novel framework designed to predict the pathogenicity of missense variants within intrinsically disordered regions (IDRs). The workflow first defines IDR locations and missense variants using data from MobiDB and ClinVar. Subsequently, wild-type and mutant sequences are processed through ALBATROSS and ProtTrans to extract global conformational (gIDRc), phase separation propensity (PS), and protein language model embedding features.

In the part of feature preprocessing, global average pooling compresses the embeddings, followed by F-statistic scoring to reduce dimensionality from 2048 to the top 20 features. The authors compare four computational strategies to optimize the input embeddings. After evaluating four classifiers, XGBoost demonstrated superior performance and was fine-tuned using Optuna. Model performance was further enhanced by incorporating prediction scores from AlphaMissense, ESM1b, and EVE as auxiliary features.

For validation, Mann-Whitney U tests and SHAP value analyses confirm the discriminative power of key features and the model's interpretability. Benchmarking against 23 existing models in dbNSFP reveals that the proposed framework, especially when integrating AlphaMissense, EVE, and ESM1b scores, achieves state-of-the-art performance. Furthermore, analysis based on GnomAD allele frequencies supports the biological validity of the predictions.

2.Specific Comments

1.The manuscript employs distinct processing strategies for different feature types: while ProtTrans embeddings were subjected to four different combination methods (L1, L2, Average, Hadamard), the biophysical features (gIDRc and PS) were processed solely using absolute delta change.

It is implied that biophysical features primarily reflect structural stability disruption, where the magnitude of change correlates with pathogenicity. However, since protein embeddings also encode structural and functional information, the rationale for not exploring similar complex interaction terms for biophysical features is unclear. If possible, briefly explaining why similar complex calculations weren’t attempted for features(PS and gIDRc) would help readers better understand the innovation of the feature processing strategy.

2.The manuscript demonstrates performance improvements achieved by individually adding prediction scores from AlphaMissense, EVE, and ESM1b as a new feature. To further evaluate the independent contributions and synergistic effects of these external features, would incorporating the following comparative experiments strengthen the evaluation section: (1) assessing model performance using only the scores of these three external predictors (without the authors' proposed features) as input, and (2) evaluating performance when the scores of all three models are all used as input features. This would help clarify whether the improvement is primarily driven by a single powerful model (such as AlphaMissense) or stems from the complementary fusion of multi features.

3.For the "Evaluation of dbNSFP predictors with functions" in the part of Results, where 23 models from the dbNSFP database were evaluated on classifying missense variants, could you specify which dataset of missense variants was used for this evaluation? Was it the test set?

4.The figures in the manuscript have low clarity, making them difficult to read. If all figures could be replaced with high-clarity vector graphics (such as EPS, or SVG formats), it would allow data details to be clearly displayed during reading.

5.Currently, the description of feature processing in the manuscript structure is relatively fragmented: the delta change processing for PS and gIDRc is introduced in the "Model generation" section, while the four computational methods for embedding are detailed in the "Data preprocessing" section. It would enhance the coherence of the article if all content of Feature preprocessing (including the delta change for physical features and the four computational strategies for embedding) could be consolidated into a single section for a unified introduction. Alternatively, briefly previewing the processing workflow for embedding in the "Model generation" section would also improve readability.

6.Figure 1C illustrates the processing workflow for WT and Mutant embeddings. If I understand correctly, the current figure may not fully reflect the compression process from "sequence embeddings matrix to single vector." If convenient to modify, it is suggested to more intuitively display this embeddings of sequence change in the figure: explicitly label the embeddings as an L*2048 matrix (L is the amino acid sequence length), which becomes a 1*2048 vector after Average Pooling. This will help readers understand the features embeddings transformation process more quickly.

**Reviewer #3:**  Authors developed a machine learning model to evaluate clinical significance of missense variants in the intrinsic disordered regions (IDR). They showed that combinations of their model with either of features arising from ALBATROSS, IDR-relevant compositional features, or ProtTrans efficiently predicted pathogenic variants in IDR. Additional analyses are required. In addition, the results are not appropriately presented in the manuscript.

1. The manuscript is not clearly written and words are used inconsistently. Readers need to know in advance what kinds of information were included in each dataset or generated by each tool. Clearly state which features in which dataset or tool were used, and how these features were used in the analysis. The Feature Generation section starting from line 190 may include the required information. Reorganize the manuscript.

2. Abstract. Words representing feature names used in the abstract are different from those in the manuscript. Use consistent words throughout the manuscript.

3. Introduction. Reviewer does not understand what authors hope to indicate by showing variants in HBB and FGFR3. The phenotypic difference arises from the difference in the variant genes. These examples have no relevance to the current study.

4. Introduction. References 6 to 11 are insufficient. There are more than 30 similar models. At least commonly used models should be included in the references.

5. Introduction. “However, a large portion of missense variants remain unclassified due to the rarity in the population.” This statement is confusing. Rarity is not the cause of uncertainty.

6. Figure 1 shows that the ALBATROSS score, the IDR-relevant compositional features, and the ProtTrans score were used to make a binary model to classify disease-causing IDR variants. This scheme is against what are explained in the abstract or the manuscript.

7. Figure 1C. Biopython is not addressed in the manuscript or the legend.

8. Figure 1C. “Average” of ProtTrans is difficult to understand. This is also applicable to the Methods. Why was averaging able to show the difference between the wild-type and mutant IDRs?

9. Model generation and other sections. How were IDRs selected? Authors repeatedly state that IDRs were selected by AlphaFold-RSA, but they also state that IDRs are taken from MobiDB. If AlphaFold-RSA was used, indicate their thresholds or categories. If MobiDB was used, which categories were recognized as IDRs.

10. Line 163. Give a rationale why the threshold of 0.581 was chosen. Also explain what this score is.

11. Line 177. Why the variants were largely reduced from 15,999 to 2,203. If VUSs were excluded indicate as such.

12. Line 182. ClinVar variants were mapped to dbNSFP. The statement is opposite.

13. Line 182. Explain why the number of variants was reduced from 2203 to 2104. dbNSFP is a database of saturation mutagenesis and should include all available missense variants.

14. Line 200. Reviewer supposes that “2048-dimensional embeddings for both mutant and WT sequences for every amino acid” should be “2048-dimensional embeddings for every amino acid in both mutant and WT sequences”. This kind of incorrect statements make this manuscript difficult to understand. If a reader does not know what ProtTrans generates, the reader will be confused.

15. Line 201. How were the embeddings averaged? The dimensions were reduced from 2048 to 1 for each amino acid or the 2048 dimensions remained unchanged but each score was averaged along the entire amino acids. Which is correct?

16. Line 204. The process to reduce the dimension to 20 is not clearly stated. In line 201, authors state 1 single embedding. In addition, feature elimination process always causes overfitting. Explain in detail how the features were eliminated and show that testing dataset was not touched in the process of feature elimination.

17. Lines 246, 257, 290 and others. AUC of what? ROC-AUC, PR-AUC, or something else?

18. Suppl. Fig. S2. Indicate which models used ClinVar and HGMD, and which models were unsupervised.

19. Legend for Figure 3A. Reviewer does not understand how and where square root was applied. In line 331, square root was applied to four features. How about Scaling Exponent?

20. Legend for Figure 3B. AUC is not indicated in the figure. ROC is shown in the figure.

21. Line 346. Reviewer supposes that the four combination methods were applied to embeddings of ProtTrans. Clarify this. Otherwise, the story is difficult to follow. In addition, gIDRc and PS used in Figure 4 are not always used in other figures or in the manuscript. This discrepancy causes confusion.

22. Legend for Figure 4A. “Accuracy performance” is inaccurate. Authors are showing ROC-AUC.

23. Legend for Figure 4B. Clarify that PR-AUC is indicated.

24. Figure 4. Show statistical significance by two-way ANOVA or Kruskal-Wallis test with an appropriate post hoc test.

25. Line 376. Show the effect of HPO on ROC-AUC. PR-AUC is not sufficient.

26. Line 379. Show ROC-AUC.

27. Line 380. Leakage by checking exact sequences should not be included in Results. Authors do not even have to address this in Materials and Methods, although this should be carefully scrutinized when making a dataset.

28. S3 Fig. What is the last number 631 in the best feature in the SHAP score? Lack of clear explanation in the method prevents the reviewer understand what is presented in this figure.

29. Line 414 and others. Always indicate ROC-AUC when PR-AUC is indicated.

30. Fig. 5. Baseline is shown three times and vertical scales are different in three panels. Make a single graph with baseline, EVE alone, ESM1b alone, AlphaMissense alone, EVE enhanced, ESM1b enhanced, and AlphaMissense enhanced, so that the performances of these models can be easily recognized.

31. Legend for Fig. 5. Multiple comparisons are not adjusted for.

32. S4 Fig. Show statistical significance.

33. Section starting from line 463 and S5 Fig. Showing only favorable variants is not fair and misleading. Remove this section. If authors hope to keep this section, show variants that resulted in unfavorable prediction outcomes in the authors’ models.

34. Section starting from line 472. A statement “EVE Enhanced showing the most pronounced bimodal distribution” cannot be recognized in S6 Fig or anywhere else.

35. Authors are showing that the combination of their tool with either AlphaMissense, ESM1b, or EVE markedly improved the performance. Why were all the three previously reported scores not added the authors’ model? Reviewer also hope to know whether the combination of the three previous scores outperformed the authors’ model alone.

36. Variants with high minor allelic frequency (MAF) can be easily recognized as benign variants. We do not have to use any tool for such variants. Excluding these variants would make a balanced dataset with similar numbers of benign and mutant variants. Reviewer suggests making models after excluding these high MAF variants.

**********

6. PLOS authors have the option to publish the peer review history of their article (what does this mean?). If published, this will include your full peer review and any attached files.

If you choose “no”, your identity will remain anonymous but your review may still be made public.

**Do you want your identity to be public for this peer review?**  For information about this choice, including consent withdrawal, please see our Privacy Policy.

Reviewer #1: No

Reviewer #2: No

Reviewer #3: No

**********

[NOTE: If reviewer comments were submitted as an attachment file, they will be attached to this email and accessible via the submission site. Please log into your account, locate the manuscript record, and check for the action link "View Attachments". If this link does not appear, there are no attachment files.]

To ensure your figures meet our technical requirements, please review our figure guidelines: https://journals.plos.org/plosone/s/figures

You may also use PLOS’s free figure tool, NAAS, to help you prepare publication quality figures: https://journals.plos.org/plosone/s/figures#loc-tools-for-figure-preparation.

NAAS will assess whether your figures meet our technical requirements by comparing each figure against our figure specifications.

# Author comment: Author response to Decision Letter 1

**DOI:** 10.1371/journal.pone.0354365.r002

28 Apr 2026

Response to reviewers attached as PDF

Attachment Submitted filename: PLOS_reviewer_response.pdf

# Peer review: Decision Letter 1

**DOI:** 10.1371/journal.pone.0354365.r003

**Authors:** Tomoyoshi Komiyama

20 May 2026

PONE-D-26-04061R1

Enhancing Missense Variant Classification in Predicted Intrinsically Disordered Regions.

PLOS One

Dear Dr. Hart,

Thank you for submitting your manuscript to PLOS ONE. After careful consideration, we feel that it has merit but does not fully meet PLOS ONE’s publication criteria as it currently stands. Therefore, we invite you to submit a revised version of the manuscript that addresses the points raised during the review process.

Please submit your revised manuscript by Jul 04 2026 11:59PM. If you will need more time than this to complete your revisions, please reply to this message or contact the journal office at plosone@plos.org. When you're ready to submit your revision, log on to https://www.editorialmanager.com/pone/ and select the 'Submissions Needing Revision' folder to locate your manuscript file.

Please include the following items when submitting your revised manuscript:

- A letter that responds to each point raised by the academic editor and reviewer(s). You should upload this letter as a separate file labeled 'Response to Reviewers'.
- A marked-up copy of your manuscript that highlights changes made to the original version. You should upload this as a separate file labeled 'Revised Manuscript with Track Changes'.
- An unmarked version of your revised paper without tracked changes. You should upload this as a separate file labeled 'Manuscript'.

If you would like to make changes to your financial disclosure, please include your updated statement in your cover letter. Guidelines for resubmitting your figure files are available below the reviewer comments at the end of this letter.

If applicable, we recommend that you deposit your laboratory protocols in protocols.io to enhance the reproducibility of your results. Protocols.io assigns your protocol its own identifier (DOI) so that it can be cited independently in the future. For instructions see: https://journals.plos.org/plosone/s/submission-guidelines#loc-laboratory-protocols. Additionally, PLOS ONE offers an option for publishing peer-reviewed Lab Protocol articles, which describe protocols hosted on protocols.io. Read more information on sharing protocols at https://plos.org/protocols?utm_medium=editorial-email&utm_source=authorletters&utm_campaign=protocols.

As the corresponding author, your ORCID iD is verified in the submission system and will appear in the published article. PLOS supports the use of ORCID, and we encourage all coauthors to register for an ORCID iD and use it as well. Please encourage your coauthors to verify their ORCID iD within the submission system before final acceptance, as unverified ORCID iDs will not appear in the published article. *Only*  the individual author can complete the verification step; PLOS staff *cannot*  verify ORCID iDs on behalf of authors.

We look forward to receiving your revised manuscript.

Kind regards,

Tomoyoshi Komiyama, Ph.D

Academic Editor

PLOS One

Journal Requirements:

1. If the reviewer comments include a recommendation to cite specific previously published works, please review and evaluate these publications to determine whether they are relevant and should be cited. There is no requirement to cite these works unless the editor has indicated otherwise.

2. Please review your reference list to ensure that it is complete and correct. If you have cited papers that have been retracted, please include the rationale for doing so in the manuscript text, or remove these references and replace them with relevant current references. Any changes to the reference list should be mentioned in the rebuttal letter that accompanies your revised manuscript. If you need to cite a retracted article, indicate the article’s retracted status in the References list and also include a citation and full reference for the retraction notice.

**Additional Editor Comments:**

Dear authors,

Thank you for submitting your revised manuscript.

I think it is an improvement from the previous revision.

However, one reviewer had additional questions.

Please answer these questions as listed below.

Tomoyoshi Komiyama

[Note: HTML markup is below. Please do not edit.]

Reviewers' comments:

Reviewer's Responses to Questions

**Comments to the Author**

1. If the authors have adequately addressed your comments raised in a previous round of review and you feel that this manuscript is now acceptable for publication, you may indicate that here to bypass the “Comments to the Author” section, enter your conflict of interest statement in the “Confidential to Editor” section, and submit your "Accept" recommendation.

Reviewer #2: All comments have been addressed

Reviewer #3: (No Response)

**********

2. Is the manuscript technically sound, and do the data support the conclusions?

The manuscript must describe a technically sound piece of scientific research with data that supports the conclusions. Experiments must have been conducted rigorously, with appropriate controls, replication, and sample sizes. The conclusions must be drawn appropriately based on the data presented.

Reviewer #2: Yes

Reviewer #3: Yes

**********

3. Has the statistical analysis been performed appropriately and rigorously?

Reviewer #2: Yes

Reviewer #3: Yes

**********

4. Have the authors made all data underlying the findings in their manuscript fully available?

The PLOS Data policy requires authors to make all data underlying the findings described in their manuscript fully available without restriction, with rare exception (please refer to the Data Availability Statement in the manuscript PDF file). The data should be provided as part of the manuscript or its supporting information, or deposited to a public repository. For example, in addition to summary statistics, the data points behind means, medians and variance measures should be available. If there are restrictions on publicly sharing data—e.g. participant privacy or use of data from a third party—those must be specified.

Reviewer #2: Yes

Reviewer #3: Yes

**********

5. Is the manuscript presented in an intelligible fashion and written in standard English?

PLOS ONE does not copyedit accepted manuscripts, so the language in submitted articles must be clear, correct, and unambiguous. Any typographical or grammatical errors should be corrected at revision, so please note any specific errors here.

Reviewer #2: Yes

Reviewer #3: Yes

**********

6. Review Comments to the Author

Please use the space provided to explain your answers to the questions above. You may also include additional comments for the author, including concerns about dual publication, research ethics, or publication ethics. (Please upload your review as an attachment if it exceeds 20,000 characters)

Reviewer #2: (No Response)

Reviewer #3: The reviewer appreciates the authors’ efforts to comply with the reviewer’s concerns. Most of the concerns have been answered and amended. However, the reviewer still has the following concerns.

1. Line 51. A statement on K24N in TP53 still does not fit to this section. Even if K24N is in IDR, the relevance of IDR has not been addressed up to this point. In addition, a reference is missing for this statement. Eliminate this statement. Alternatively, show an example of two different amino acid substitutions at an identical amino acid resulted in mild and severe phenotypes.

2. Line 55. “However, a large proportion of missense variants remain classified as VUS, in part because many occur at low population frequencies that preclude robust statistical evidence for or against pathogenicity.” Low MAF is “in favor of” pathogenicity, and should not be “against” pathogenicity. The reviewer supposes that the authors hope to state that low MAF alone is not sufficient to conclude that VUS is pathogenic.

3. Line 56. Spell out VUS in its first occurrence.

4. Line 61. “These models employ machine and deep learning techniques trained on common variants observed in non-human primates as a proxy.” Most models have been trained on variants in humans and not in non-human primates. Some models take evolutional information, but most are not restricted non-human primates. If authors are confident, clearly indicate which models were based on non-human primates and which were on humans.

**********

7. PLOS authors have the option to publish the peer review history of their article (what does this mean?). If published, this will include your full peer review and any attached files.

If you choose “no”, your identity will remain anonymous but your review may still be made public.

**Do you want your identity to be public for this peer review?**  For information about this choice, including consent withdrawal, please see our Privacy Policy.

Reviewer #2: No

Reviewer #3: No

**********

[NOTE: If reviewer comments were submitted as an attachment file, they will be attached to this email and accessible via the submission site. Please log into your account, locate the manuscript record, and check for the action link "View Attachments". If this link does not appear, there are no attachment files.]

To ensure your figures meet our technical requirements, please review our figure guidelines: https://journals.plos.org/plosone/s/figures

You may also use PLOS’s free figure tool, NAAS, to help you prepare publication quality figures: https://journals.plos.org/plosone/s/figures#loc-tools-for-figure-preparation.

NAAS will assess whether your figures meet our technical requirements by comparing each figure against our figure specifications.

# Author comment: Author response to Decision Letter 2

**DOI:** 10.1371/journal.pone.0354365.r004

1 Jul 2026

Response to Reviewer 3

We thank the reviewer for their comments on the manuscript. We have addressed all four comments as detailed below.

Comment 1: Line 51. Line 51. A statement on K24N in TP53 still does not fit to this section. Even if K24N is in IDR, the relevance of IDR has not been addressed up to this point. In addition, a reference is missing for this statement. Eliminate this statement. Alternatively, show an example of two different amino acid substitutions at an identical amino acid resulted in mild and severe phenotypes.

Response: The TP53 K24N example was premature at this point in the Introduction, as intrinsically disordered regions had not yet been introduced. The statement has been removed. In its place, we have substituted it with “For example, the Glu6Val substitution causes sickle cell disease through polymerization of deoxygenated hemoglobin, while the Glu6Lys substitution results in hemoglobin C disease, a clinically milder condition characterized by hemolytic anemia without the vaso-occlusive complications characteristic of sickle cell disease”, which illustrates how different amino acid substitutions at the same position can produce markedly different clinical severities. A reference has been added for this example.

Comment 2: Line 55. “However, a large proportion of missense variants remain classified as VUS, in part because many occur at low population frequencies that preclude robust statistical evidence for or against pathogenicity.” Low MAF is “in favor of” pathogenicity and should not be “against” pathogenicity. The reviewer supposes that the authors hope to state that low MAF alone is not sufficient to conclude that VUS is pathogenic.

Response: The original sentence was misleading. It has been revised to, "However, a large proportion of missense variants remain classified as variants of uncertain significance (VUS), in part because many occur at low population frequencies that, while consistent with pathogenicity under purifying selection, are insufficient alone to establish a causal role in disease, and because functional characterization of individual variants at scale remains impractical." This accurately reflects that low allele frequency is consistent with pathogenicity but not sufficient to confirm it.

Comment 3: Line 56. Spell out VUS in its first occurrence.

Response: VUS is now spelled out as "variants of uncertain significance (VUS)" at its first occurrence in the revised manuscript.

Comment 4: Line 61. “These models employ machine and deep learning techniques trained on common variants observed in non-human primates as a proxy.” Most models have been trained on variants in humans and not in non-human primates. Some models take evolutional information, but most are not restricted non-human primates. If authors are confident, clearly indicate which models were based on non-human primates and which were on humans.

Response: The original sentence incorrectly implied that most models are trained exclusively on non-human primate variants. The sentence has been revised to accurately distinguish training approaches. We updated the introduction with the following:

Many of these models are trained on human variant datasets, including population databases and clinical repositories such as ClinVar and gnomAD. A subset leverage evolutionary signals derived from cross-species sequence alignments, while PrimateAI specifically uses common variants observed in non-human primates as a proxy for benign variation, under the assumption that variants tolerated across primate species are unlikely to be pathogenic in humans

Attachment Submitted filename: PLOS_ONE.ResponsetoReviewer3.docx

# Peer review: Decision Letter 2

**DOI:** 10.1371/journal.pone.0354365.r005

**Authors:** Tomoyoshi Komiyama

7 Jul 2026

Enhancing Missense Variant Classification in Predicted Intrinsically Disordered Regions.

PONE-D-26-04061R2

Dear Dr. Hart,

We’re pleased to inform you that your manuscript has been judged scientifically suitable for publication and will be formally accepted for publication once it meets all outstanding technical requirements.

Within one week, you’ll receive an e-mail detailing the required amendments. When these have been addressed, you’ll receive a formal acceptance letter and your manuscript will be scheduled for publication.

An invoice will be generated when your article is formally accepted. Please note, if your institution has a publishing partnership with PLOS and your article meets the relevant criteria, all or part of your publication costs will be covered. Please make sure your user information is up-to-date by logging into Editorial Manager at Editorial Manager® and clicking the ‘Update My Information' link at the top of the page. For questions related to billing, please contact billing support.

If your institution or institutions have a press office, please notify them about your upcoming paper to help maximize its impact. If they’ll be preparing press materials, please inform our press team as soon as possible -- no later than 48 hours after receiving the formal acceptance. Your manuscript will remain under strict press embargo until 2 pm Eastern Time on the date of publication. For more information, please contact onepress@plos.org.

Kind regards,

Tomoyoshi Komiyama, Ph.D

Academic Editor

PLOS One

Additional Editor Comments (optional):

Dear Authors,

Thank you for submitting your revised manuscript.

I found the revised version much easier to understand than the original manuscript.

I am satisfied with both your responses to the reviewers' comments and the revisions you have made.

Therefore, I am pleased to recommend acceptance of this manuscript.

I believe this manuscript will be of considerable interest to the journal's readers.

Tomoyoshi Komiyama

Reviewers' comments:

Reviewer's Responses to Questions

**Comments to the Author**

1. If the authors have adequately addressed your comments raised in a previous round of review and you feel that this manuscript is now acceptable for publication, you may indicate that here to bypass the “Comments to the Author” section, enter your conflict of interest statement in the “Confidential to Editor” section, and submit your "Accept" recommendation.

Reviewer #3: All comments have been addressed

**********

2. Is the manuscript technically sound, and do the data support the conclusions?

The manuscript must describe a technically sound piece of scientific research with data that supports the conclusions. Experiments must have been conducted rigorously, with appropriate controls, replication, and sample sizes. The conclusions must be drawn appropriately based on the data presented.

Reviewer #3: Yes

**********

3. Has the statistical analysis been performed appropriately and rigorously?

Reviewer #3: N/A

**********

4. Have the authors made all data underlying the findings in their manuscript fully available?

The PLOS Data policy requires authors to make all data underlying the findings described in their manuscript fully available without restriction, with rare exception (please refer to the Data Availability Statement in the manuscript PDF file). The data should be provided as part of the manuscript or its supporting information, or deposited to a public repository. For example, in addition to summary statistics, the data points behind means, medians and variance measures should be available. If there are restrictions on publicly sharing data—e.g. participant privacy or use of data from a third party—those must be specified.

Reviewer #3: Yes

**********

5. Is the manuscript presented in an intelligible fashion and written in standard English?

PLOS ONE does not copyedit accepted manuscripts, so the language in submitted articles must be clear, correct, and unambiguous. Any typographical or grammatical errors should be corrected at revision, so please note any specific errors here.

Reviewer #3: Yes

**********

6. Review Comments to the Author

Please use the space provided to explain your answers to the questions above. You may also include additional comments for the author, including concerns about dual publication, research ethics, or publication ethics. (Please upload your review as an attachment if it exceeds 20,000 characters)

Reviewer #3: (No Response)

**********

7. PLOS authors have the option to publish the peer review history of their article (what does this mean?). If published, this will include your full peer review and any attached files.

If you choose “no”, your identity will remain anonymous but your review may still be made public.

**Do you want your identity to be public for this peer review?**  For information about this choice, including consent withdrawal, please see our Privacy Policy.

Reviewer #3: No

**********

# Editor report: Acceptance letter

**DOI:** 10.1371/journal.pone.0354365.r006

**Authors:** Tomoyoshi Komiyama

PONE-D-26-04061R2

PLOS One

Dear Dr. Hart,

I'm pleased to inform you that your manuscript has been deemed suitable for publication in PLOS One. Congratulations! Your manuscript is now being handed over to our production team.

At this stage, our production department will prepare your paper for publication. This includes ensuring the following:

* All references, tables, and figures are properly cited

* All relevant supporting information is included in the manuscript submission,

* There are no issues that prevent the paper from being properly typeset

You will receive further instructions from the production team, including instructions on how to review your proof when it is ready. Please keep in mind that we are working through a large volume of accepted articles, so please give us a few days to review your paper and let you know the next and final steps.

Lastly, if your institution or institutions have a press office, please let them know about your upcoming paper now to help maximize its impact. If they'll be preparing press materials, please inform our press team within the next 48 hours. Your manuscript will remain under strict press embargo until 2 pm Eastern Time on the date of publication. For more information, please contact onepress@plos.org.

You will receive an invoice from PLOS for your publication fee after your manuscript has reached the completed accept phase. If you receive an email requesting payment before acceptance or for any other service, this may be a phishing scheme. Learn how to identify phishing emails and protect your accounts at https://explore.plos.org/phishing.

If we can help with anything else, please email us at customercare@plos.org.

Thank you for submitting your work to PLOS ONE and supporting open access.

Kind regards,

PLOS ONE Editorial Office Staff

on behalf of

Dr. Tomoyoshi Komiyama

Academic Editor

PLOS One
